# cNTnC and fYTnC2, Genetically Encoded Green Calcium Indicators Based on Troponin C from Fast Animals

**DOI:** 10.3390/ijms232314614

**Published:** 2022-11-23

**Authors:** Oksana M. Subach, Anna V. Vlaskina, Yuliya K. Agapova, Dmitriy A. Korzhenevskiy, Alena Y. Nikolaeva, Anna M. Varizhuk, Maksim F. Subach, Maxim V. Patrushev, Kiryl D. Piatkevich, Konstantin M. Boyko, Fedor V. Subach

**Affiliations:** 1 Complex of NBICS Technologies, National Research Center “Kurchatov Institute”, Moscow 123182, Russia; 2 Laboratory of Electrophysiology, Federal Center of Brain Research and Neurotechnologies, Ostrovityanova Str. 1, Bld. 10, Moscow 125367, Russia; 3 Center for Precision Genome Editing and Genetic Technologies for Biomedicine, Federal Research and Clinical Center of Physical-Chemical Medicine of Federal Medical Biological Agency, Malaya Pirogovskaya Str. 1a, Moscow 119435, Russia; 4 Moscow Institute of Physics and Technology, Dolgoprudny 141701, Russia; 5 Department of Chemistry, Lomonosov Moscow State University, Moscow 119991, Russia; 6 School of Life Sciences, Westlake University, Hangzhou 310024, China; 7 Westlake Laboratory of Life Sciences and Biomedicine, Hangzhou 310024, China; 8 Institute of Basic Medical Sciences, Westlake Institute for Advanced Study, Hangzhou 310024, China; 9 Bach Institute of Biochemistry, Research Center of Biotechnology of the Russian Academy of Sciences, Leninsky Ave. 33, Bld. 2, Moscow 119071, Russia

**Keywords:** genetically encoded green calcium indicators, protein engineering, fluorescence imaging, cNTnC, fYTnC2, green fluorescent protein

## Abstract

NTnC-like green fluorescent genetically encoded calcium indicators (GECIs) with two calcium ion binding sites were constructed using the insertion of truncated troponin C (TnC) from *Opsanus tau* into green fluorescent proteins (GFPs). These GECIs are small proteins containing the N- and C-termini of GFP; they exert a limited effect on the cellular free calcium ion concentration; and in contrast to calmodulin-based calcium indicators they lack undesired interactions with intracellular proteins in neurons. The available TnC-based NTnC or YTnC GECIs had either an inverted response and high brightness but a limited dynamic range or a positive response and fast kinetics in neurons but lower brightness and an enhanced but still limited dF/F dynamic range. Here, we solved the crystal structure of NTnC at 2.5 Å resolution. Based on this structure, we developed positive NTnC2 and inverted iNTnC2 GECIs with a large dF/F dynamic range in vitro but very slow rise and decay kinetics in neurons. To overcome their slow responsiveness, we swapped TnC from *O. tau* in NTnC2 with truncated troponin C proteins from the muscles of fast animals, namely, the falcon, hummingbird, cheetah, bat, rattlesnake, and ant, and then optimized the resulting constructs using directed molecular evolution. Characterization of the engineered variants using purified proteins, mammalian cells, and neuronal cultures revealed cNTnC GECI with truncated TnC from *Calypte anna* (hummingbird) to have the largest dF/F fluorescence response and fast dissociation kinetics in neuronal cultures. In addition, based on the insertion of truncated TnCs from fast animals into YTnC2, we developed fYTnC2 GECI with TnC from *Falco peregrinus* (falcon). The purified proteins cNTnC and fYTnC2 had 8- and 6-fold higher molecular brightness and 7- and 6-fold larger dF/F responses to the increase in Ca^2+^ ion concentration than YTnC, respectively. cNTnC GECI was also 4-fold more photostable than YTnC and fYTnC2 GECIs. Finally, we assessed the developed GECIs in primary mouse neuronal cultures stimulated with an external electric field; in these conditions, cNTnC had a 2.4-fold higher dF/F fluorescence response than YTnC and fYTnC2 and was the same or slightly slower (1.4-fold) than fYTnC2 and YTnC in the rise and decay half-times, respectively.

## 1. Introduction

Genetically encoded calcium indicators (GECIs) are valuable tools for neuronal imaging in cells, tissues, and whole organisms [1]. Intensiometric GECIs with green fluorescence are the most common. Green GECIs consist of green fluorescent protein (GFP) and Ca^2+^-binding components. Calmodulin (CaM) in combination with the M13 peptide from myosin light chain kinase (CaM/M13) or a minimal Ca^2+^-binding motif from the C-terminal domain of troponin C (TnC) can serve as the calcium ion binding component [2]. The latter has a smaller size (approximately 100 amino acid residues less than the CaM/M13 pair) and two Ca^2+^-binding sites instead of four sites in the CaM/M13 pair. Since genes encoding GECIs are often delivered into model organisms by insertion into adeno-associated virus (AAV) vectors and since the packaging capacity of AAV particles is limited, smaller GECIs are preferred. A lower number of Ca^2+^-binding sites is preferable because of the more linear response of a sensor to changes in calcium ion concentration and the smaller influence on intracellular free Ca^2+^ ion concentration (as GECI may serve as Ca^2+^ buffer) [3].

The use of the C-terminal domain of TnC instead of the CaM/M13 pair as a Ca^2+^-binding component may have the additional advantage of a lack of undesired interactions with the intracellular environment. The vast majority of GECIs are based on mammalian CaM and M13 peptide, for example, the widespread GCaMP family. There is evidence that because of the mammalian CaM/M13 pair the unregulated expression of GCaMP-derived GECIs caused cardiomegaly in adult transgenic mice when expressed in mouse heart cells [4] and resulted in cytotoxicity and death of neurons in mice [5,6], abnormal GCaMPs accumulation in the nuclei of neurons [7,8], aberrant epileptiform neuronal activity in some genotypes of transgenic mice [9], and perturbation of the gating of the Cav1/CaM complex [8] in neurons. We previously showed that due to CaM’s lower amino acid sequence homology to metazoa orthologs, the utilization of CaMs from *Aspergillus niger* fungus in FGCaMP and FGCaMP7 GECIs and from *Schizosaccharomyces pombe* fungus in FRCaMP GECI prevented undesired interactions with proteins inside mammalian cells. Another way to prevent such interactions is the application of other calcium ion binding proteins. Calmodulin is a major Ca^2+^-binding protein in the brain, but troponin C plays a key role as a Ca^2+^-binding protein in skeletal and cardiac muscles. Therefore, troponin C should have less influence on the intracellular environment in neurons.

Recently, two GECIs constructed by the insertion of truncated troponin C from the swim bladder and white muscle of *Opsanus tau* into mNeonGreen (NTnC) [10] or YFP (YTnC) [11] GFPs were reported. This design allowed the maintenance of intact N- and C-termini of GFP, which is favorable for tagging any cellular protein [11]. NTnC with an inverted phenotype had a major disadvantage, namely, a low ∆F/F fluorescence response of 100% between its Ca^2+^-free and Ca^2+^-saturated states. YTnC had a positive response, low molecular brightness, and an improved but still limited ∆F/F fluorescence response 2.9-fold higher than that of NTnC. To overcome the limitations of the current troponin-based GECIs, we decided to use truncated versions of troponin C from the muscles of several other fast animals as calcium-binding domains for the development of GECIs.

Herein, the aim of our work was to develop the NTnC-like green fluorescent GECIs having two calcium ion binding sites with enhanced brightness and fluorescence responses in neurons. To achieve this purpose, we solved the X-ray structure of the NTnC calcium indicator. Subsequent structure-guided and random mutagenesis strategies were applied to NTnC to engineer the NTnC2 and iNTnC2 GECIs. However, they had slow on- and off-kinetics in neurons. To speed up the kinetics of NTnC2, we swapped troponin C from *O. tau* with troponins from animals with fast muscles, namely, the falcon, hummingbird, cheetah, bat, rattlesnake, and ant. Linker optimization followed by several rounds of random mutagenesis and characterization of purified proteins allowed us to choose the best four GECIs with troponins from *Calypte anna* (hummingbird), *Acinonyx jubatus* (cheetah), and *Falco peregrinus* (falcon). The characterization of the chosen mutants in mammalian cells and neuronal cultures showed that cNTnC GECI with troponin from *Calypte anna* (hummingbird) had the largest ∆F/F fluorescence response and fast dissociation kinetics in neuronal cultures. In addition, in an attempt to speed up the neuronal kinetics, we developed TnC-based GECIs with an EYFP fluorescent domain and troponins from falcon, hummingbird, and cheetah. Characterization in vitro and in mammalian cells demonstrated that fYTnC2 with troponin from *Falco peregrinus* (falcon) had the largest dF/F fluorescence response. In vitro, cNTnC and fYTnC2 outperformed YTnC GECI by 8- and 6-fold in molecular brightness, respectively, by 7- and 6-fold in ∆F/F fluorescence response, and by 4- and 4-fold in photobleaching half-time. In neuronal cultures with electrical stimulation, cNTnC surpassed fYTnC and YTnC by 2.4-fold in the ∆F/F fluorescence response and had the same on/off kinetics as fYTnC and 1.4-fold longer rise- and decay half-times compared to YTnC.

## 2. Results and Discussion

### 2.1. NTnC Crystal Structure

**Figure 1:**
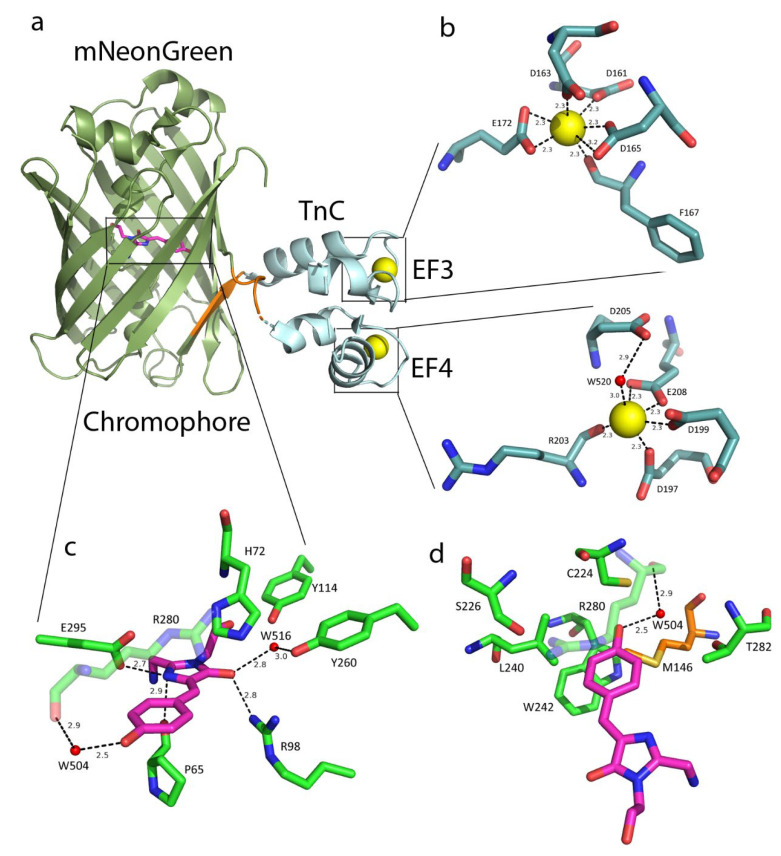
Crystal structure of the NTnC indicator in the Ca^2+^-bound state (PDB 5mwc). (**a**) The overall structure of NTnC. TnC, troponin C. Ca^2+^-binding motifs 3 and 4 (EF3 and EF4). (**b**) Ca^2+^ ions and water molecules are represented as yellow and red spheres, respectively; the numbering corresponds to the NTnC sequence. Hydrogen bonds are shown as dashed lines with corresponding distances. (**c**,**d**) Chromophore (magenta) with its surrounding environment (green amino acids). Panel (**d**) is rotated by 90° compared to Panel (**c**). Residues around the phenolic group are depicted. M146 is shown in orange.

To improve the fluorescence response of NTnC in neurons, the crystal structure of the NTnC indicator with two bound calcium ions was obtained at 2.45 Å resolution (Table S1 and Figure 1). NTnC has two domain structures consisting of covalently linked fluorescent and Ca^2+^-binding domains (Figure 1a). The fluorescent domain has a typical β-barrel fold with the GFP-like ^68^GYG^70^ chromophore in the central α-helix, and the Ca^2+^-binding domain consists of two EF-hands characteristic of truncated troponin C.

Analysis of the crystal structure of the NTnC sensor made it possible to identify amino acid residues close to the chromophore. The chromophore in NTnC makes three direct H-bonds with P65, R98, and E295 as well as two water-mediated H-bonds with Y260 and R280 (Figure 1c). The phenolic hydroxyl group of the chromophore forms one water-mediated hydrogen bond with the main carboxyl of R280. In addition, R280 is stacked with the phenolic group of the chromophore. The chromophore in NTnC has a *cis* configuration. Usually, in this configuration, the phenolic hydroxyl group of the chromophore contacts the amino acid at position 148 (enumeration to EGFP). In the NTnC crystal structure, position 148 corresponds to C224, which is close to the chromophore, but the SH-group of this cysteine is oriented away from the chromophore, and the distance between the SH-group of C224 and the OH-group of the chromophore is significantly larger than the H-bond distance. Instead, in the crystal structure of NTnC (with two Ca^2+^ ions bound), the methyl group of the M146 residue in Linker 1 is near the OH-group of the chromophore. We can assume that in the Ca^2+^-free state of the indicator, the C224 residue in mNeonGreen stabilizes the deprotonated state of the Y69 residue of the chromophore, blocking the access of the solvent to the chromophore and providing a high fluorescence intensity of the NTnC indicator. When Ca^2+^ ions bind, the distance between troponin C and mNeonGreen β-barrel decreases, and the M146 residue in Linker 1 displaces the C224 residue. In addition to the M146 and C224 residues, the L240, R280, and T282 amino acids are close to the phenolic hydroxyl group (Figure 1c,d).

Having analyzed the crystal structure of the NTnC indicator, we determined that the first Ca^2+^ ion in NTnC is coordinated by five amino acids in the EF3 motif: D161 (+x), D163 (+y), D165 (+z), F167 (−y), and E172 (−z), and the second Ca^2+^ ion is also coordinated by five amino acid residues in the EF4 motif: D197 (+x), D199 (+y), R203 (−y), D205 through the solvent molecule (−x) and E208 (−z) (Figure 1b). Thus, using the crystal structure of NTnC, we identified both the amino acid residues close to the chromophore and the amino acid residues coordinating calcium ions.

### 2.2. Development and Characterization of NTnC2 with Troponin C from Toadfish

To improve the fluorescence response of NTnC based on crystallographic data, we engineered a rational library with randomization of three amino acids close to the phenolic hydroxyl group of the chromophore, namely, M146X/C224X/L240X. In this library, we found clones exhibiting both direct and reverse phenotypes with ∆F/F responses of 170% and 450%, respectively, which were 1.7- and 4.5-fold larger than the 100% ∆F/F response of the original NTnC. Thus, with the help of directed mutagenesis based on the crystal structure of NTnC, we were able to increase the fluorescence response of the NTnC sensor and find both positive and inverted phenotypes.

**Figure 2:**
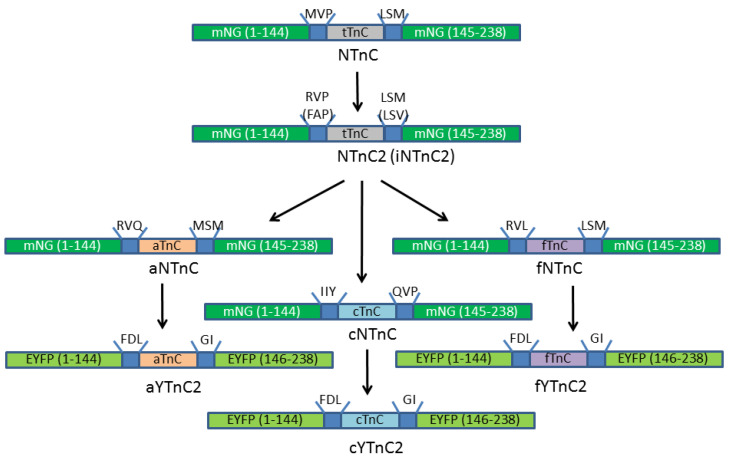
Scheme of engineering NTnC-like indicators.

Mutants of NTnC with positive and inverted phenotypes obtained as a result of rational mutagenesis were subjected to eight and six rounds of random mutagenesis, respectively, followed by screening of bacterial colonies on Petri dishes to increase both brightness and fluorescence response to Ca^2+^. The ∆F/F fluorescence responses of the best positive and inverted sensors NTnC2 and iNTnC2 achieved 90- and 30-fold increases, respectively (Figure 2 and Figure S1 and Table 1). According to stopped-flow fluorimetry data, the NTnC2 mutant had 8- and 3.8-fold slower association and dissociation rates with calcium ions, respectively, than the control GCaMP6s (Table 1). For the iNTnC2 mutant, the dissociation rate was 14-fold slower. Hence, based on the crystallographic data for the NTnC indicator, we succeeded in developing versions with a significantly improved ∆F/F fluorescence response but with significantly slower calcium association/dissociation kinetics.

### 2.3. Development and Characterization of Calcium Indicators Based on mNeonGreen and Troponin C from Fasting Animals

To speed up the kinetics of calcium association–dissociation for NTnC-like indicators, we decided to swap truncated troponin C from toadfish with truncated troponins C from muscles of fast animals. We chose troponin C from *Acinonyx jubatus* (cheetah), *Calypte anna* (hummingbird), *Falco peregrinus* (falcon), *Crotalus adamanteus* (rattlesnake), *Harpegnathos saltator* (ant), and *Myotis lucifugus* (bat) (Figure S2).

Then, based on NTnC2 GECI, we created libraries of calcium indicators with the synthetized truncated troponin C genes and randomized the amino acid residues located in linkers between the fluorescent and Ca^2+^-binding domains (in each linker, we randomized one amino acid close to troponin C, Figure S3). We suggest that unlike the NTnC indicator, whose chromophore is in the *cis* configuration (Figure 1), the chromophore of the NTnC2 sensor is in the *trans* configuration, since L and D residues at the 224 and 240 positions (similar to the 148 and 165 positions in EGFP) should stabilize the *trans*-configuration of the chromophore. As a result of screening libraries on Petri dishes under a fluorescent stereomicroscope followed by the analysis of bacterial lysates on a plate reader in 96-well format, we found variants with a positive response to an increase in Ca^2+^ ion concentration. The largest ∆F/F fluorescence response upon binding to Ca^2+^ ions (from 1.4- to 5.8-fold) was observed for fNTnC#18trans, aNTnC#2trans, and cNTnC#9trans indicators containing troponins from falcon, cheetah, and hummingbird, respectively. These mutants were chosen for random mutagenesis.

Since NTnC2 and iNTnC2 indicators with a *trans*-chromophore had slow calcium association–dissociation rates, unlike the NTnC indicator with a *cis*-chromophore, we reasoned that a *cis*-chromophore might be favorable for faster Ca^2+^ binding/dissociation. Based on this assumption, we constructed libraries of calcium indicators (Figure S4) based on NCaMP4, the brightest indicator from the NCaMP series with a *cis*-chromophore [14] and truncated troponin C molecules from falcon, hummingbird, and cheetah, which showed the largest contrast for indicators with a *trans*-chromophore. Both linkers consisting of three amino acid residues between the fluorescent and calcium-binding regions were randomized in each library (Figure S4). From each library, we selected clones with the largest contrast and highest positive response to the addition of calcium ions. The largest ∆F/F fluorescence responses to calcium ions (varying from 1.8- to 18-fold) were observed for fNTnC#9cis, aNTnC#9cis, and cNTnC#10cis indicators containing troponins from falcon, cheetah, and hummingbird, respectively. These mutants were selected for further random mutagenesis.

To increase the ∆F/F dynamic range and brightness of the selected calcium indicators, we carried out 2–9 rounds of random mutagenesis. The selected variants were subjected to random mutagenesis followed by screening on Petri dishes and bacterial lysates, as described above. In the case of cNTnCcis, aNTnCcis, and fNTnCcis indicators with a *cis*-chromophore, we performed 2, 4, and 9 rounds of random mutagenesis, respectively. For cNTnCtrans, aNTnCtrans, and fNTnCtrans indicators with a *trans*-chromophore, we performed 4, 4, and 5 rounds of random mutagenesis, respectively. As a result, six improved versions of these indicators were obtained: fNTnCtrans, fNTnCcis, cNTnCtrans, cNTnCcis, aNTnCtrans, and aNTnCcis (Table 2, Figures S5 and S6). According to the alignment of amino acid residues for *trans*-mutants (Figure S5), aNTnCtrans, cNTnCtrans, and fNTnCtrans GECIs contain 10, 14, and 12 mutations compared to the original libraries, respectively. Of these, 2, 5, and 3 for aNTnCtrans; 3, 8, and 3 for cNTnCtrans; and 2, 4, and 6 for fNTnC#trans are in the linker, fluorescent, and Ca^2+^-binding parts, respectively. According to the alignment of amino acid residues for *cis* mutants (Figure S6), aNTnCcis, cNTnCcis, and fNTnCcis GECIs contain 13, 11, and 21 mutations, respectively, compared to the original libraries. Of these, 6, 4, and 3 for aNTnCcis; 6, 3, and 2 for cNTnCcis; and 6, 12, and 3 for fNTnCcis are in the linker, fluorescent, and Ca^2+^-binding regions, respectively.

The ∆F/F fluorescence responses and dissociation constants (Kd) of selected mutants upon binding to calcium ions were characterized using lysates of bacterial cells expressing mutants. The ∆F/F fluorescence responses of mutants in vitro were improved up to 20–137-fold (Table 2). Kd in the presence of 1 mM Mg^2+^ ions (conditions resembling the concentration of magnesium ions in mammalian cells) ranged from 288 to 721 nM (Table 2). These Kd values are in the same range as the Kd values of widely used calcium indicators that reliably reflect changes in the physiological concentration of calcium ions in the cytosol of mammalian cells, such as GCaMP6s and GCaMP6f [7], whose Kd values in the presence of 1 mM Mg^2+^ are 227 and 632 nM, respectively. The calcium affinity for the developed indicators was optimal for monitoring changes in calcium concentration during neuronal activity, which typically ranges from 50–100 nM at rest to 250–10,000 nM during activation in the cytosol of mammalian cells [15].

**Figure 3:**
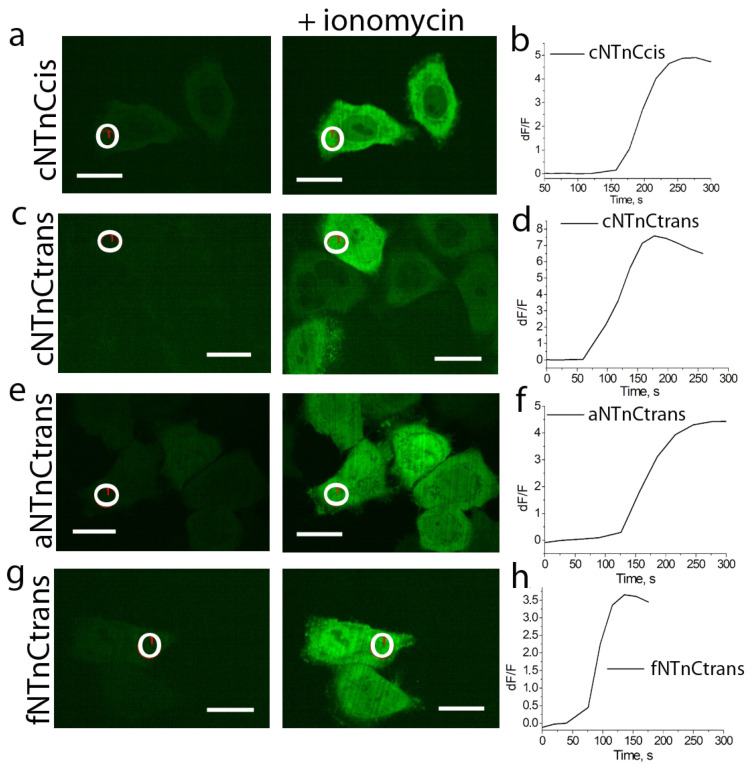
Response of NTnC-like variants with truncated troponin C from hummingbird (**a**–**d**), cheetah (**e**,**f**), and falcon (**g**,**h**) to Ca^2+^ elevation in HeLa cells induced by ionomycin addition. Left, confocal images of HeLa cells expressing GECIs in the green fluorescence channel (488 nm excitation) before and after ionomycin addition. Right, the graphs show the time-lapse changes in green fluorescence as a result of the addition of 5 μM ionomycin. The graphs illustrate the changes in green fluorescence in the areas indicated with white circles. Scale bars, 200 μm.

The next step was the characterization of the developed TnC-based indicators in HeLa mammalian cells. For this purpose, we expressed the fNTnCtrans, fNTnCcis, cNTnCcis, cNTnCtrans, aNTnCtrans, and aNTnCcis indicators in the cytosol of HeLa cells and studied their response to the ionomycin-induced increase in calcium concentration. After the addition of 2.5 μM ionomycin, we observed an increase in green fluorescence upon excitation with 488 nm light (Figure 3). It should be noted that the expression of indicators was observed both in the cytosol and in the nucleus of HeLa cells; we suggest that due to the small size of the TnC-based indicators, they enter the cellular nucleus by passive diffusion. The average ∆F/F fluorescence responses obtained in HeLa cells and related to Ca^2+^ concentration increase are shown in Table 2. The ∆F/F fluorescence responses in HeLa cells decreased 2–171-fold compared to proteins purified from bacterial cells. Unfortunately, the aNTnCtrans indicator with the largest ∆F/F response as protein isolated from bacterial cells showed the smallest response when expressed in HeLa cells. For further testing in neuronal cultures, fNTnCtrans, cNTnCcis, cNTnCtrans, and aNTnCtrans indicators with the largest ∆F/F fluorescence response in HeLa cells were selected.

**Figure 4:**
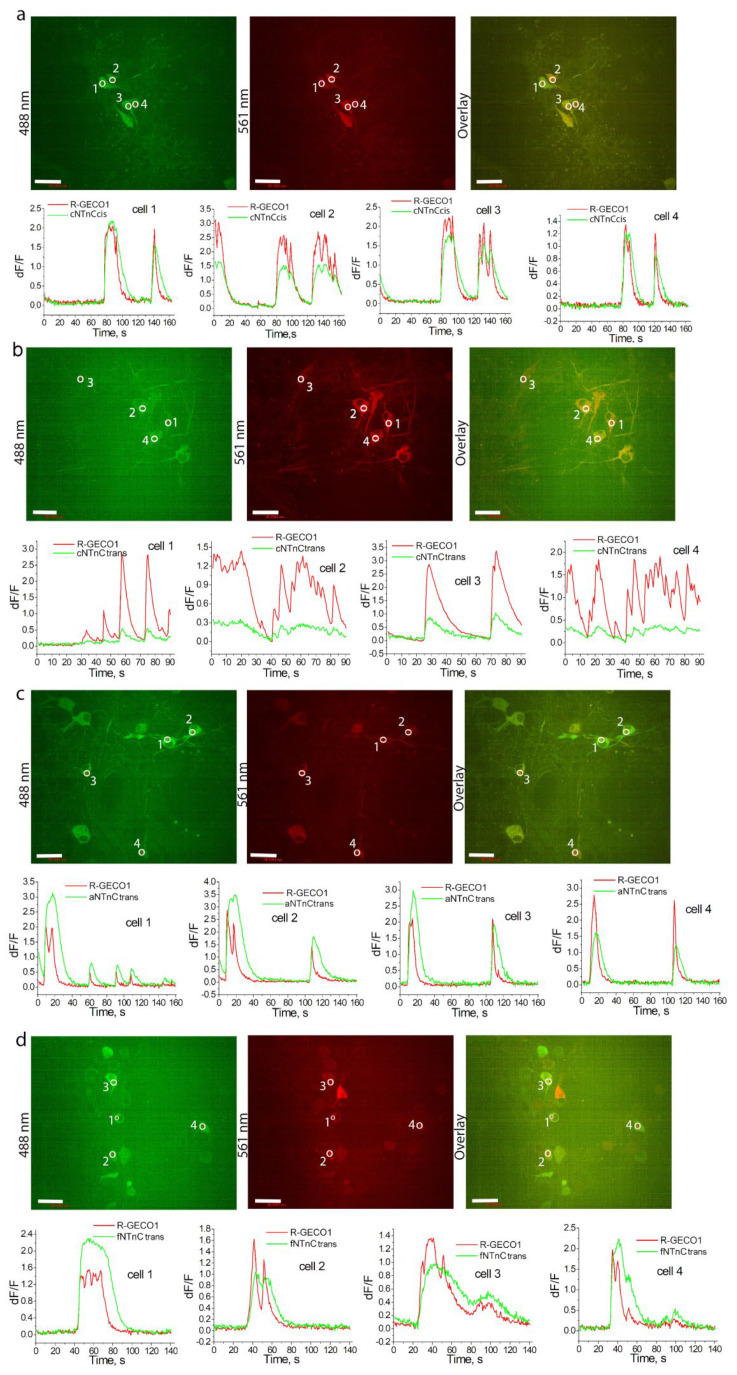
Spontaneous neuronal activity of NTnC-like variants with truncated troponin C from hummingbird (**a**,**b**), cheetah (**c**), and falcon (**d**) coexpressed with R-GECO1 in neuronal cultures. Top, confocal images of HeLa cells coexpressing green GECIs with red R-GECO1 indicator in green and red fluorescence channels (excitation at 488 and 561 nm, respectively) and their overlay. Bottom, the graphs for each mutant show the changes in green and red fluorescence for four cells in the areas indicated with white circles. Scale bars, 50 μm.

We then tested the applicability of the fNTnCtrans, cNTnCcis, cNTnCtrans, and aNTnCtrans indicators for monitoring spontaneous (nonspecific) neuronal activity in cultured neuronal cells and compared their performance with each other and with the coexpressed red R-GECO1 indicator. When coexpressed with the red R-GECO1 indicator in the cytosol of dissociated neuronal cultures, green indicators responded to spontaneous neuronal activities (Figure 4). We evaluated the ∆F/F fluorescence responses and decay half-times for each mutant compared to R-GECO1 (Table 2). cNTnCtrans demonstrated the dramatic 320-fold drop in the fluorescence response upon transition from expression in bacterial cells to expression in neuronal culture. All other three GECIs showed a similar 35–41-fold decrease in fluorescence response upon transition to neuronal culture. The cNTnCcis indicator was the best. It had a similar ∆F/F response and a 2-fold longer decay half-time compared to R-GECO1. Thus, of all indicators based on mNeonGreen and truncated troponin C molecules from fast animals, we chose the cNTnCcis indicator, henceforth called cNTnC, for detailed characterization in vitro and in neuronal cultures.

### 2.4. Engineering of Calcium Indicators Composed of EYFP and Truncated Troponins C from Fast Animals

We noted that calcium indicators based on mNeonGreen fluorescent protein such as NTnC [10] had slower kinetics in neurons than indicators based on EYFP fluorescent protein such as YTnC [11]. In neuronal cultures, the developed cNTnCcis indicator had a decay half-time 2-fold longer than that for R-GECO1 (Table 2). To speed up the kinetics of NTnC-like indicators, we decided to swap the mNeonGreen fluorescent part with EYFP. We designed calcium indicators with Ca^2+^-binding troponin C components from the best NTnC-derived fNTnCtrans, cNTnCcis, and aNTnCtrans indicators and fluorescent components with linkers from YTnC2 (bright YTnC variant, unpublished data) (Figure 2). After one round of random mutagenesis followed by screening on Petri dishes and in bacterial lysates, we found fYTnC2, cYTnC2, and aYTnC2 variants with ∆F/F responses varying from 4 to 10 and Kd values varying from 564 to 709 nM measured using purified proteins in vitro (Table 3 and Figure S7).

We also expressed the developed YTnC-derived indicators in HeLa cells. When switching from bacterial expression in vitro of the YTnC-derived GECIs to their expression in mammalian cells, a 1.7–12.5-fold drop in the fluorescence response was observed (Table 3). The maximal averaged ∆F/F fluorescence response to ionomycin-induced Ca^2+^ increase was observed for fYTnC2 (Table 3 and Figure S8). Therefore, in addition to cNTnC GECI, we decided to characterize fYTnC2 in detail in vitro and in neuronal cultures.

### 2.5. Properties of cNTnC and fYTnC2 GECIs In Vitro

**Figure 5:**
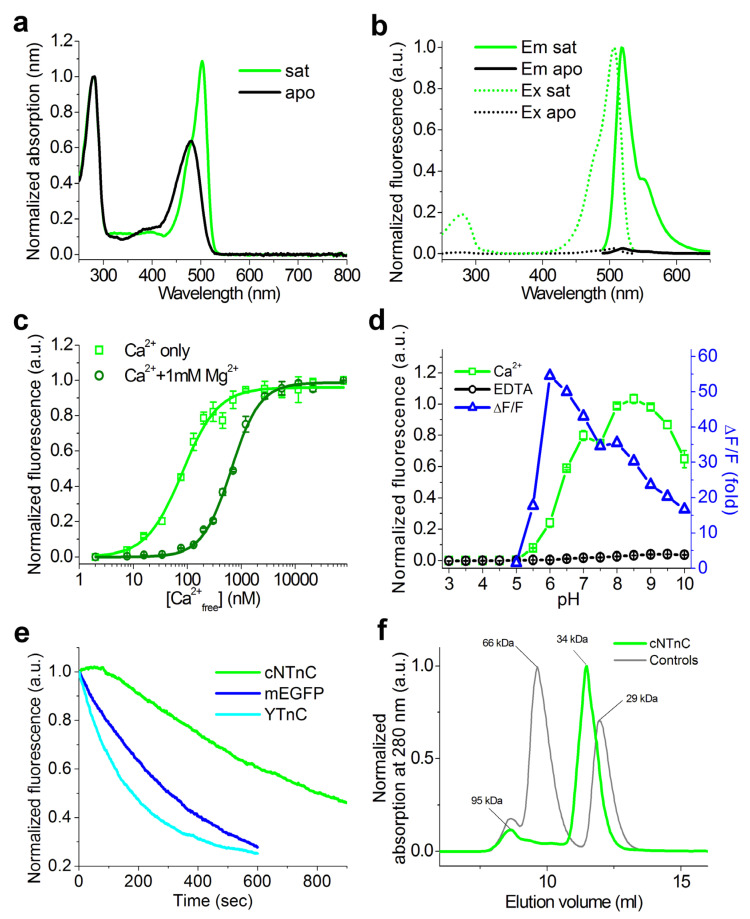
In vitro properties of purified cNTnC protein. (**a**) Absorbance spectra of cNTnC in Ca^2+^-free (apo) and Ca^2+^-bound (sat) states. (**b**) Excitation and emission spectra of cNTnC in Ca^2+^-free and Ca^2+^-bound states. (**c**) Ca^2+^ titration curves of cNTnC in the absence or presence of 1 mM MgCl_2_. (**d**) Intensity and dynamic range of cNTnC as a function of pH. The ΔF/F fluorescence response (fold) at each pH value was determined as the ratio of cNTnC fluorescence intensity in the absence of Ca^2+^ to that in the presence of Ca^2+^. (**e**) Photobleaching curves for cNTnC and YTnC in the Ca^2+^-bound states and for mEGFP. (**f**) Fast protein liquid chromatography of cNTnC. cNTnC was eluted in 20 mM Tris-HCl (pH 7.80) and 200 mM NaCl buffer. The molecular weight of cNTnC was calculated from a linear regression of the dependence of the logarithm of the control molecular weights vs. elution volume (Figure S10). Error represents the standard error of the estimate for the average of three records.

We next determined the biophysical properties of the cNTnC and fYTnC2 indicators. First, we measured the absorption, excitation, and emission spectra of the cNTnC and fYTnC2 proteins (Figure 5a,b and Figure S9a,b). In both Ca^2+^-bound and Ca^2+^-free (apo) states, cNTnC had absorption/excitation/emission maxima at 480–503/506/518–520 nm, which are characteristic of the anionic form of the green/yellow chromophore (Table 4). In the apo- and saturated states, cNTnC was nonfluorescent and fluorescent, respectively. The fYTnC2 indicator in the Ca^2+^-free state had absorption/excitation/emission peaks at 414/416/516 nm, which are characteristic of the protonated form of the green/yellow chromophore, and was fluorescent (Table S2). In the Ca^2+^-saturated state, it existed in both protonated and anionic forms with absorption maxima at 406 and 494 nm, whereas the anionic form was mainly fluorescent with excitation/emission peaks at 498/518 nm. Hence, the cNTnC and fYTnC2 indicators were intensiometric and ratiometric, respectively.

Then, we characterized the brightness of the developed cNTnC and fYTnC2 indicators (Table 4 and Table S2). In the Ca^2+^-saturated state, cNTnC and fYTnC2 were 7.6- and 5.6-fold brighter than YTnC, respectively. In the apo-state at 488 excitation, cNTnC and fYTnC2 were 39- and 19-fold less fluorescent than in the Ca^2+^-saturated state, respectively. In the apo-state, fYTnC2 was fluorescent at 405 nm excitation and demonstrated 4-fold less brightness than in the Ca^2+^-saturated state. Unlike the cNTnC indicator, fYTnC2 can be treated as a ratiometric GECI with fluorescence in both the apo- and saturated states. The addition of 1 mM Mg^2+^ ions decreased the ∆F/F response of the cNTnC and fYTnC2 indicators by 1.97- and 1.06-fold, respectively; 1 mM Mg^2+^ ions decreased the ∆F/F response of the YTnC indicator by 3.6-fold. Therefore, purified cNTnC and fYTnC2 proteins in the Ca^2+^-bound state were brighter than YTnC by a factor of 6–8, and their ∆F/F responses were less affected by the presence of Mg^2+^ ions.

Next, to understand how variations in pH in different cellular compartments may influence the fluorescence responses of the cNTnC and fYTnC2 indicators, we characterized the pH dependencies of their fluorescence in the Ca^2+^-bound and Ca^2+^-free states. In the presence of 100 μM calcium ions, the cNTnC indicator reached 80% of its fluorescence with a pKa value of 6.2 and 100% of its fluorescence with a pKa of 7.8 (Figure 5d). The fluorescence of cNTnCapo exhibited pH dependence with a pKa value of 7.4. Different pH sensitivities of cNTnCsat and cNTnCapo caused a dependence of the cNTnC ∆F/F fluorescence response on pH. fYTnC2, identical to the control YTnC in the apo state, had two pH transitions with pKa values of 5.2 and 8.2, and in the saturated state, it had only one pKa value of 6.3 (Figure S9d). These differences in pH sensitivity for both forms resulted in a strong dependence of the fYTnC2 ∆F/F fluorescence response on pH. Hence, the ∆F/F fluorescence responses of both cNTnC and fYTnC2 GECIs were affected by pH variations.

Two important characteristics of GECIs are their affinity to calcium ions and ∆F/F fluorescence response. To estimate these parameters, we further measured the equilibrium binding dependences of cNTnC and fYTnC2 fluorescence on Ca^2+^ ion concentrations in the absence and presence of 1 mM Mg^2+^ ions (close to the 0.5–1.5 mM Mg^2+^ ion concentrations estimated in the cytosol of mammalian cells [16,17]). In the absence of Mg^2+^ ions, cNTnC and fYTnC2 had Kd values of 81 and 477 nM that were 2.8-fold smaller and 2.1-fold larger than the Kd of YTnC, respectively (Table 4 and Table S2, Figure 5c and Figure S9c). The ∆F/F fluorescence responses of the cNTnC and fYTnC2 indicators were 3.5- and 1.6-fold larger than the ∆F/F response for YTnC GECI, respectively. In the presence of Mg^2+^ ions, the Kd values of cNTnC and fYTnC2 GECIs were 651 and 709 nM, which were 1.6- and 1.7-fold larger than the Kd value for YTnC GECI, respectively (Table 4 and Table S2, Figure 5c and Figure S9c). The ∆F/F fluorescence responses of the cNTnC and fYTnC2 GECIs in these conditions were approximately 6-fold larger than the ∆F/F response for the YTnC indicator. Thus, in vitro, the developed cNTnC and fYTnC2 indicators outperformed YTnC in ∆F/F fluorescence responses. The affinities of the developed indicators were in the range of the physiological free Ca^2+^ ion concentration changes from 50–100 nM to 250–10,000 nM observed in the cytosol of mammalian cells [15]. The affinity of cNTnC to Ca^2+^ ions strongly depended on the concentration of magnesium ions, since it differed by a factor of 8 in the absence and presence of Mg^2+^ ions. Hence, both in the absence and in the presence of Mg^2+^ ions, the cNTnC and fYTnC2 indicators demonstrated affinity to calcium ions suitable for their application in the cytosol of mammalian cells, and both cNTnC and fYTnC2 indicators demonstrated ∆F/F responses 6-fold larger than the ∆F/F response for the YTnC indicator.

After that, we characterized the one-photon photostability of the purified cNTnC and fYTnC2 proteins (Figure 5e and Figure S9e). We used suspensions of microdroplets of these proteins in oil and irradiated them with a metal halide lamp using 470/40 nm light and a 63× oil objective lens. cNTnC GECI was 4.2-fold more photostable than YTnC, whereas fYTnC2 GECI had similar photostability to YTnC (Table 4 and Figure S6e). Therefore, the cNTnC indicator was substantially 4-fold more photostable than the YTnC and fYTnC2 GECIs.

We also estimated the maturation rates of cNTnC and fYTnC2 at 37 °C (Table 3 and Table S2). The maturation half-times of cNTnC and fYTnC2 were 4.8-fold longer and 3.5-fold faster than that for the YTnC control protein.

We next assessed the oligomeric state of cNTnC and fYTnC2 GECIs as dimers and tetramers in contrast to monomers, whose tendency to aggregate may cause cytotoxicity and hinder their application for protein tagging in mammalian cells [18,19]. We analyzed the purified cNTnC and fYTnC2 proteins using fast protein liquid chromatography (FPLC) (Figure 5f, Figures S9f and S10). Both proteins eluted mostly as monomers with a small admixture of dimers, while fYTnC2 contained more dimers. Hence, both developed GECIs were mostly monomers and might be appropriate for protein labeling. Overall, both cNTnC and fYTnC2 outperformed the YTnC indicator in most of the important characteristics studied.

### 2.6. Validation of cNTnC and fYTnC2 Indicators in Neuronal Cultures

To evaluate the functionality of cNTnC and fYTnC2 GECIs in neurons, we characterized their fluorescence changes during spontaneous and electrically stimulated activity in primary mouse neuronal cultures. To measure spontaneous activity, we cotransduced neuronal cultures with rAAV particles carrying CAG-NES-cNTnC or CAG-NES-fYTnC2 together with rAAVs carrying CAG-NES-R-GECO1 and recorded the spontaneous activity of neurons between 12 and 19 days in vitro (DIVs) (Figure 4a and Figure S11). Both cNTnC and fYTnC2 GECIs showed even distribution throughout neurons, including nuclei, soma, and branches. R-GECO1 demonstrated a fluorescent signal only in soma and branches excluding nuclei. This difference may be attributed to the larger size of R-GECO1 on more than 100 amino acids. The average ∆F/F responses of the cNTnC and fYTnC2 GECIs were 1.25- and 2-fold lower than that of R-GECO1 (Figure S11b). 

The average rise half-times for cNTnC and fYTnC2 were 1.3- and 1.2-fold longer than for R-GECO1, respectively (Figure S11c). The average decay half-times for cNTnC and fYTnC2 were 2.4- and 1.3-fold longer than that for R-GECO1, respectively (Figure S11d). Therefore, according to the registration of spontaneous neural activity, cNTnC had a 1.6-fold larger ∆F/F fluorescence response but a 1.9-fold longer decay half-time than the fYTnC2 indicator.

**Figure 6:**
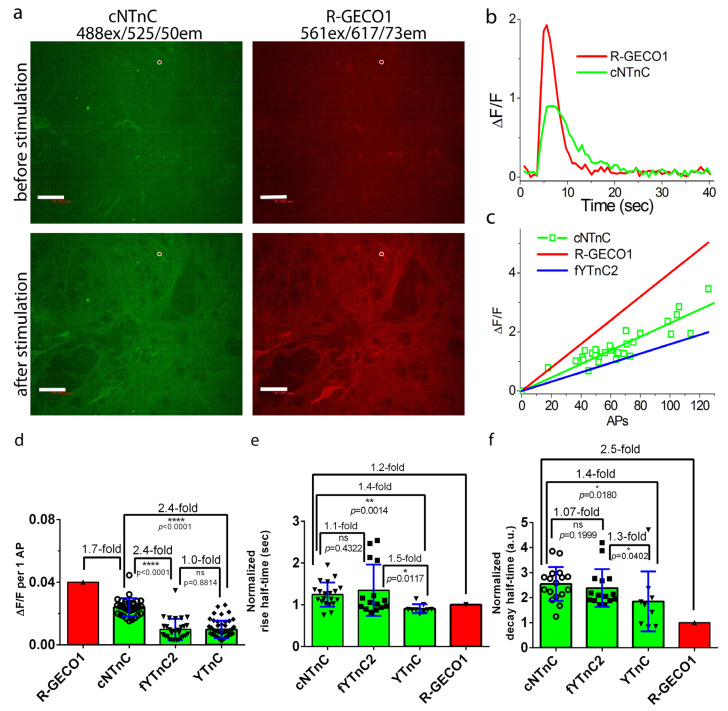
Comparison of the responses of green cNTnC and red R-GECO1 indicators to the external field stimulation of neurons coexpressing GECIs in dissociated neuronal cultures. Neuronal cultures coexpressing the NES-cNTnC and NES-R-GECO1 indicators were imaged and stimulated on DIV 21–22. Neuronal cultures were transduced on DIV 4 with a mixture of rAAVs carrying NES-cNTnC and NES-R-GECO1. (**a**) Confocal images of neuronal culture coexpressing the NES-cNTnC and NES-R-GECO1 indicators before (top) and after (bottom) electrical stimulation. Scale bar, 50 μm. (**b**) The graph illustrates ΔF/F changes in the green and red fluorescence of the cNTnC and R-GECO1 indicators in response to electrical field stimulation. The changes on the graph correspond to the area indicated in Panel a as a white circle. (**c**) The dependence of ΔF/F responses of the cNTnC indicator vs. the number of action potentials (APs). The number of APs was determined according to the ΔF/F response of the R-GECO1 indicator (0.04 per 1 AP) coexpressed in the same cell and assuming that the response’s linearity in the examined AP range (linear fitting for cNTnC had R2 value of 0.89935). The dependences of ΔF/F responses on APs for fYTnC2 and R-GECO1 were added to compare the results. (**d**) The ΔF/F responses per AP for cNTnC and fYTnC2 were calculated according to the ΔF/F response of R-GECO1 (0.04 per 1 AP) in the same cell. For comparison, the ΔF/F response per AP for YTnC was added from previous work [11]. (**e**,**f**) The rise (**e**) and decay (**f**) half-times of cNTnC and fYTnC2. (**d**–**f**) Error bars are the standard deviations across 31–49 cells. Ns, not significant, *p* > 0.05. *, *p* value is from 0.01 to 0.05. **, *p* value is from 0.001 to 0.01. ****, *p* < 0.0001.

To validate the ∆F/F responses and the rise and decay half-times of the developed TnC-derived GECIs in detail in neurons and to compare these characteristics with those of the YTnC GECI, we used an external electric field to stimulate dissociated neuronal cultures coexpressing green cNTnC or fYTnC2 GECIs, together with the red R-GECO1 indicator (Figure 6a and Figure S12a). For YTnC, we referred to corresponding characteristics in neurons obtained earlier [11]. The ∆F/F response per 1 action potential (AP) of cNTnC (2.4 ± 0.6%) was 2.4-fold larger (*p* < 0.0001) than the respective responses for fYTnC2 (1 ± 0.7%) and YTnC (1 ± 0.6%) (Figure 6b–d and Figure S12b,c). The rise half-times of the cNTnC (1.2 ± 0.3) and fYTnC2 (1.3 ± 0.6) indicators were similar to each other and were 1.4–1.5-fold longer than the rise half-time for YTnC (0.9 ± 0.1) (Figure 6e). The decay half-times for the cNTnC (2.5 ± 0.7) and fYTnC2 (2.4 ± 0.8) indicators were also similar to each other and were 1.3–1.4-fold longer than the rise half-time for YTnC (1.8 ± 1.2) (Figure 6f). Thus, among all TnC-based GECIs with two Ca^2+^ ion binding sites, cNTnC demonstrated the best characteristics for the visualization of electrical-field-evoked activity in cultured neurons.

## 3. Materials and Methods

### 3.1. Protein Crystallization

An initial crystallization screening of NTnC was performed using a robotic crystallization system (Rigaku, Austin, TX, USA) and commercial 96-well crystallization screens (Hampton Research, Aliso Viejo, CA, USA) at 15 °C utilizing the sitting drop vapor diffusion method. The protein concentration was 15 mg/mL in the following buffer: 20 mM Tris-HCl, 250 mM NaCl, pH 8.0, 5 mM CaCl_2_. The initial conditions were optimized using the hanging-drop vapor-diffusion method in 24-well VDX plates. Rod-like crystals were grown within 4 weeks in the following conditions: 0.1 M sodium acetate, 22.5% PEG 3350, 10% PEG 400, and 5% DMSO.

### 3.2. Data Collection, Processing, Structure Solution, and Refinement

NTnC crystals were briefly soaked in a cryosolution containing precipitant supplemented with 10% DMSO (Hampton Research, Aliso Viejo, CA, USA) immediately prior to diffraction data collection and flash-frozen in liquid nitrogen. The X-ray data were collected from a single crystal at 100 K at beamline ID30-A of the European Synchrotron Radiation Facility (Grenoble, France) [21]. The data were indexed, integrated, and scaled using the XDS program [22] (Table S1). The program Pointless [23] suggested the 4_1_2_1_2 space group.

The structure was solved using the molecular replacement method using MOLREP [24], and the structure of the Yellow–Green Fluorescent Protein mNeonGreen protein (PDB ID 5LTR) was used as an initial model. Structure refinement was carried out using the REFMAC5 program of the CCP4 suite [25]. The visual inspection of electron density maps and the manual rebuilding of the model were carried out using the COOT interactive graphics program [26]. The hydrogen atoms in fixed positions were introduced during the refinement. In the final model, an asymmetric unit contained two independent copies of the protein with chromophores, solvent molecules, and calcium ions.

### 3.3. Structure Analysis and Validation

The visual inspection of the structure was carried out using COOT and the PyMOL Molecular Graphics System, Version 1.9.0.0 (Schrödinger, New York, NY, USA). Structure comparison and superposition were performed using the PDBeFold program [27], while contacts were analyzed using PDBePISA [28].

### 3.4. Cloning of Bacterial Vectors, Mutagenesis, and Library Screening

NTnC-like indicators were cloned into the pBAD/HisB plasmid at BglII/EcoRI restriction sites using the Neon-BglII-2/Neon-EcoRI-r2 (cNTnC) and Fw-LSSmOrange-BglII/Rv-GFP-EcoRI (fYTnC2) primers (Evrogen, Moscow, Russia) listed in Table S3 to express NTnC-like indicators in BW25113 bacterial cells.

Site-directed libraries for rational mutagenesis of the parental NTnC protein at three positions were generated using the Fw-M146NNS, Rv-M146NNS, Rv-C224NNS, and Fw-L240NNS primers listed in Table S3. The whole gene was assembled using PCR with overlapping fragments [29]. Random mutations were introduced over the whole length of the sensor gene using PCR in the presence of manganese ions with conditions to achieve 2–3 random mutations per 1000 bp (according to the Diversify PCR Random Mutagenesis Kit User Manual, Clontech, Mountain View, CA, USA). Generated libraries were inserted at BglII/EcoRI restriction sites of the pBAD/HisB-TorA-mTagBFP-sfGFP plasmid by swapping the mTagBFP gene.

Library construction and screening of bacterial libraries were performed as previously described [10]. Briefly, screening of bacterial libraries was sequentially performed on Petri dishes and in bacterial lysates in a 96-well plate format.

Primary screening of approximately 10,000–20,000 colonies of bacterial library expressing GECIs variants was performed on Petri dishes under the Leica M205FA fluorescent stereomicroscope (Leica, Wetzlar, Germany). The expression of the sensors in the colonies on Petri dishes was induced with 0.0002% arabinose for 16 h at 37°C and 24 h at room temperature (r.t.). The reaction of the sensors with calcium ions was further monitored under the Leica fluorescent stereomicroscope. Green fluorescence was registered by 480/40BP excitation and 535/40BP emission filters, respectively. Fluorescence images of Petri dishes with bacterial colonies were obtained before and after spraying the plates with 100 mM EDTA, 100 mM Na_2_HPO_4_ at pH 7.4. Images obtained were analyzed using the ImageJ software and colonies with the highest brightness and contrast were selected for further analysis. Then, 120–200 mutants were chosen for bacterial streaks on Petri dishes. The screening of streaks on Petri dishes was performed analogously to the screening of colonies.

Next, approximately 20–40 mutants selected through streaks analysis were analyzed on bacterial lysates using a 96-well ModulusTM II Microplate Reader (Turner Biosystems, USA). For this purpose, the best clones selected from Petri dishes were grown in 5 mL aliquots of LB medium containing 100 µg/mL ampicillin and 0.0002% arabinose for 12–16 h at 220 rpm and 37 °C and for 24 h at r.t. The cultures were centrifuged at 1640 g for 12 min. The cell pellets were resuspended in 150 μl of B-Per solution (Thermo Scientific, Rockford, IL, USA) containing 1 mg/mL lysosyme, and 20 u/mL DNAse I (Invitrogen, Lakewood, NJ, USA) and incubated for 20 min at 37 °C. Then, the lysates were centrifuged for 2 min at 20,000 g at 4 °C. Next, the lysates were analyzed in 96-well plates. For each mutant, 2 μl of lysate was added to 200 μL of buffer (30 mM MOPS, 100 mM KCl, pH 7.2) containing 10 mM EGTA (0 μM free Ca^2+^) or 10 mM Ca-EGTA (39 μM free Ca^2+^). A total of 1–2 clones that exhibited increased brightness and fluorescence response compared to clones from the previous round of mutagenesis were subjected to the next round of random mutagenesis.

### 3.5. Protein Purification and Characterization

Protein purification, the determination of extinction coefficients and quantum yields, pH titrations, and stopped-flow experiments were performed as described in [10]. The excitation and emission fluorescence spectra were measured with a CM2203 spectrofluorometer (Solar, Minsk, Belarus). The fluorescence intensities of the developed purified green GECIs in the Ca^2+^-free and Ca^2+^-saturated states were measured in buffer (10 mM Tris-HCl, 100 mM KCl, pH 7.2) supplemented with either 10 mM EDTA or 5 mM CaCl_2_, respectively. The fluorescence intensities of buffers without indicators were subtracted.

The photobleaching of NTnC-like indicators was measured as described [10] except for the use of suspensions of purified proteins in aqueous micro droplets in mineral oil using 1.5 µL of purified proteins dialyzed in PBS buffer at a 45 µM concentration. In total, 10 µL of mineral oil was placed on the cover glass using Zeiss Axio Imager Z2 microscope (Zeiss, Oberkochen, Germany) equipped with a 120 W mercury short-arc lamp (LEJ, Jena, Germany), a 63 × 1.4 NA oil immersion objective lens (PlanApo, Zeiss, Germany), a 470/40BP excitation filter, a FT 495 beam splitter, and 525/50BP emission filters. The fluorescence intensities were extracted from image stacks using ImageJ software. The background values around the micro droplets were subtracted. No corrections were applied to the experimental data.

Size-exclusion chromatography was performed with a SuperdexTM 75 10/300 GL column using a GE AKTA Explorer (Amersham Pharmacia, UK) FPLC System.

The maturation rate of cNTnC was determined as previously described [30].

Preparative protein purification is described in the Supplementary Methods.

### 3.6. Mammalian Plasmid Construction

To construct the pAAV-*CAG*-NES-cNTnC-NES3, pAAV-*CAG*-NES-aNTnC-NES3, and pAAV-*CAG*-NES-fNTnC-NES3 plasmids, the cNTnC, aNTnC, or fNTnC genes were PCR amplified as the BglII-EcoRI fragments using the Neon-BglII2/YTnC-NES3-r primers listed in Table S3 and swapped with the mCherry gene in the pAAV-*CAG*-NES-mCherry vector. To construct the pAAV-*CAG*-NES-cYTnC2-NES3, pAAV-*CAG*-NES-aYTnC2-NES3, and pAAV-*CAG*-NES-fYTnC2-NES3 plasmids, the cYTnC2, aYTnC2, or fYTnC2 genes were PCR amplified as BglII-EcoRI fragments using the Fw-LSSmOrange-BglII/YTnC-NES3-r primers listed in Table S3 and swapped with the mCherry gene in the pAAV-*CAG*-NES-mCherry vector.

### 3.7. Mammalian Live-Cell Imaging

Transient transfection of HeLa Kyoto cells and imaging of mammalian cells were performed as described in the Supplementary Methods. The fluorescence of GECIs in the transfected mammalian cells was acquired using a laser spinning-disk Andor XDi Technology Revolution multi-point confocal system (Andor Technology, UK) equipped with an inverted Nikon Eclipse Ti-E/B microscope (Nikon Instruments, Japan), a 75 W mercury–xenon lamp (Hamamatsu, Japan), a 60× oil immersion objective NA 1.4 (Nikon, Japan), a 16-bit Neo sCMOS camera (Andor Technology, UK), laser module Revolution 600 (Andor Technology, UK), and spinning-disk module Yokogawa CSU-W1 (Andor Technology, UK). The green fluorescence intensity of the developed green GECIs and the red fluorescence intensity of the control R-GECO1 red GECI expressed in mammalian cells were acquired using the 488 or 561 nm lasers, confocal dichroic mirror 405/488/561/640 and filter wheel, 525/50 or 617/73 emission filters, respectively. The region of interest (ROI) was chosen in the cytosol of the cell and the value of fluorescence intensity was measured using the Andor iQ3.1 software (Build Number: 7.0.0.74, Belfast, UK). The background values were subtracted for each ROI.

### 3.8. Imaging in Primary Mouse Neuronal Cultures

The rAAV particles were purified from 10 15 cm dishes, as described in the original paper [11]. The preparation of dissociated neuronal cultures, transduction of neuronal cultures with a mixture of rAAV viral particles (DJ serotype) carrying AAV-CAG-NES-R-GECO1 and AAV-CAG-NES-cNTnC, AAV-CAG-NES-aNTnC, AAV-CAG-NES-fNTnC, AAV-CAG-NES-cYTnC, AAV-CAG-NES-aYTnC, or AAV-CAG-NES-fYTnC, stimulation of neuronal cultures, and imaging were carried out as described in the Supplementary Methods. The green and red fluorescence intensities were measured as described in the previous Section 3.7.

### 3.9. Statistics

To estimate the significance of the difference between two values, we used the Mann–Whitney Rank Sum Test and provided *p* values (throughout the text in the brackets) calculated for the two-tailed hypothesis. We considered the difference significant if the *p* value was <0.05.

### 3.10. Ethical Approval and Animal Care

All methods for animal care and all experimental protocols were approved by the National Research Center “Kurchatov Institute” Committee on Animal Care (NG-1/109PR of 13 February 2020) and were performed in accordance with the Russian Federation Order Requirements N 267 M3 and the National Institutes of Health Guide for the Care and Use of Laboratory Animals. The mice were used without regard to sex.

## 4. Conclusions

In conclusion, starting from the X-ray structure of the NTnC indicator, we developed and characterized a set of calcium indicators with two calcium ion binding sites based on truncated TnCs from the muscles of fast animals and green fluorescent protein, either mNeonGreen or EYFP. Using screening on purified proteins in HeLa cells and in neuronal cultures, we chose the best mNeonGreen-based cNTnC and EYFP-based fYTnC2 indicators. The characterization of the purified proteins isolated from bacterial cells showed that cNTnC had 7.6- and 1.4-fold higher brightness, 6.7- and 1.2-fold higher ∆F/F responses to increasing Ca^2+^ concentration, and 4.2-fold higher photostability than the YTnC (published earlier) and fYTnC2 GECIs, respectively. However, cNTnC also had drawbacks, since cNTnC had a 4.8- and 16.5-fold longer maturation time than YTnC and fYTnC2, respectively, at 37 °C, and its affinity to calcium ions was strongly dependent on Mg^2+^ concentration, i.e., NTnC had Kd values of 81 and 651 nM in the absence and presence of Mg^2+^ ions, respectively. When compared using the electrically evoked stimulation of primary mouse neuronal cultures, cNTnC outperformed both the fYTnC2 and YTnC indicators by 2.4-fold in the ∆F/F fluorescence response, while it had rise and decay kinetics similar to fYTnC2 and 1.4-fold slower than YTnC GECI. Therefore, cNTnC is currently the best NTnC-like GECI with two calcium ion binding sites.

Overall, for the development of the cNTnC indicator, we tested six different truncated troponin C proteins and two green fluorescent proteins. The replacement of troponin C from *O. tau* (toadfish) with troponins from fast animals in NTnC2 accelerated the calcium binding/dissociation kinetics of mNeonGreen-based NTnC-like GECIs. The response amplitude and kinetics of the indicator in mammalian cells strongly depended on the origin of the Troponin C, suggesting the correct choice of multiple Troponins to increase the probability of successful sensor development. Compared to the EYFP-based YTnC indicator, mNeonGreen-based cNTnC was substantially brighter and demonstrated a larger ∆F/F response; however, cNTnC had slightly slower kinetics than YTnC in neurons. Hence, we observed that the main characteristics of GECIs may depend on both the calcium-binding domain and fluorescent protein domain used for its construction. The application of other bright green–yellow fluorescent proteins and TnC for the development of TnC-based GECIs with two calcium ion binding sites could help to further improve their characteristics.

## Figures and Tables

**Table 1:** Properties of NTnC2 and iNTnC2 in vitro and in neuronal cultures. ^a^ Determined at 300 nM Ca^2+^. ^b^ Data from [12]. ^c^ Data from [13]. ^d^ Data from [7]. ^e^ Data from [14].

Indicator	K_d_, nM	dF/F	K_d_, hM (Mg^2+^)	dF/F (Mg^2+^)	k^on^ _obs_ at 300 nM Ca^2+^, s^−1 a^	t_1/2_^off^, s	k^off^, s^−1^	dF/F Relative to RGECO1 in Neuronal Culture
R-GECO1	482 ^b^	15 ^b^	1138 ± 43 ^c^	21.0 ± 0.2 ^c^	ND	ND	0.752 ^b^	1.0
GCaMP6s	144 ± 3 ^d^	43 ^d^	227.3 ± 0.2 ^d^	46 ^d^	0.49 ± 0.05 ^d^	1.01 ^c^	0.69 ± 0.01 ^d^	3.2 ± 2 ^e^
NTnC2	1408 ± 51	90	1290 ± 40	48	0.06	3.84	0.194 ± 0.001	0.95 ± 0.31
iNTnC2	49 ± 1	30	30 ± 1	10	ND	14.2	0.044 ± 0.002	0.12 ± 0.06

**Table 2:** Properties of fNTnC, cNTnC, and aNTnC indicator variants. ^a^ K_d_ was measured in buffer containing 1 mM MgCl_2_.

Indicator	Purified Proteins		HeLa Cells	Neuronal Culture	
	ΔF/F	K_d_, nM [Hill] ^a^	ΔF/F	ΔF/F, Versus R-GECO1	t^1/2^_off_, Versus R-GECO1
fNTnC_trans_	20	502 [1.9]	9.3 ± 4.9	0.5 ± 0.1	3.3 ± 1.5
fNTnC_cis_	30	288 [2.2]	1.7 ± 0.5	ND	ND
cNTnC_trans_	64	571 [1.8]	11.4 ± 3.2	0.2 ± 0.1	1.9 ± 0.3
cNTnC_cis_	28	556 [1.72]	6.9 ± 0.6	0.8 ± 0.2	2.4 ± 0.5
aNTnC_trans_	33	558 [1.7]	4.4 ±2.2	0.8 ± 0.3	4.5 ± 1.6
aNTnC_cis_	137	721 [2.4]	0.8 ± 0.2	ND	ND

**Table 3:** Properties of fYTnC2, cYTnC2, and aYTnC2 mutants. ^a^ Kd was measured in buffer containing 1 mM MgCl_2_.

Indicator	Purified Proteins		HeLa Cells
	ΔF/F	K_d_, nM ^a^	ΔF/F
fYTnC2	17	709	4.0 ± 1.5
cYTnC2	4	564	2.3 ± 0.2
aYTnC2	10	620	0.8 ± 0.1

**Table 4:** In vitro properties of cNTnC compared to YTnC. ^a^ Data from [11]. Data marked with an asterisk (*) were determined in this paper. ^b^ Quantum yields (QYs) were determined at pH 7.20. EGFP (QY = 0.60 [20]) was used as the reference standard. ^c^ The extinction coefficients (ε) were determined using alkaline denaturation. ^d^ Brightness was calculated as a product of the quantum yield and extinction coefficient and normalized to the brightness of EGFP, which has an extinction coefficient of 56,000 M^−1^·cm^−1^ and a quantum yield of 0.6 [20]. ^e^ The Hill coefficient is shown in brackets. ^f^ EGFP had a maturation half-time of 14 min. ^g^ Half-time to bleaching up to 50%. One-photon photobleaching was performed under a mercury lamp with drops in oil. Standard deviations are shown. EGFP had a photobleaching half-time of 305 ± 38 s.

Properties			Proteins			
			cNTnC		YTnC ^a^	
			Apo	Sat	Apo	Sat
Absorption maximum (nm)			480	503	413	495 (405)
Excitation maximum (nm)			506	506	412, 501	502 (413)
Emission maximum (nm)			520	518	514	516 (516)
Quantum yield ^b^			0.021 ± 0.004	0.53 ± 0.03	0.012	0.19 (0.03)
ε (mM^−1^ cm^−1^) ^c^			53.8 ± 4.8	82.3 ± 3.0	28 ± 2	29 ± 3 (20 ± 2)
Brightness vs. EGFP (%) ^d^			3.4	130	1	17 (2)
ΔF/F	Purified protein	0 mM Mg^2+^	38.3 ± 7.2		10.6 ± 0.4	
		1 mM Mg^2+^	19.4 ± 0.8		2.9 ± 0.2	
	HeLa cells		4.9 ± 1.4		2.0 ± 0.4	
pKa			7.36 ± 0.24	6.24 ± 0.01 7.80 ± 0.04	5.2 ± 0.1, 8.2 ± 0.1	6.3 ± 0.1
K_d_ (nM) ^e^	0 mM Mg^2+^		81 ± 6 [n = 1.3 ± 0.1]		223 ± 10 [n = 1.4 ± 0.1]	
	1 mM Mg^2+^		651 ± 22 [n = 1.6 ± 0.1]		410 ± 19 [n = 1.7 ± 0.2]	
Maturation half-time (min) ^f^			76		16	
Photobleaching half-time (s) ^g^			803 ± 90		193 ± 36 *	

## Data Availability

The data are contained within the article or its Supplementary Materials.

## Supplementary Materials

The supporting information can be downloaded at: https://www.mdpi.com/article/10.3390/ijms232314614/s1.

## Abbreviations

MDPI	Multidisciplinary Digital Publishing Institute
RFP	Red fluorescent protein
LSSRFP	Red fluorescent protein with large Stokes shift
FP	Fluorescent protein
PBS	Phosphate-buffered saline
QY	Quantum yield
SD	Standard deviation
