# Supplementary material for: cNTnC and fYTnC2, Genetically Encoded Green Calcium Indicators Based on Troponin C from Fast Animals
Source: Int J Mol Sci. 2022 Nov 23;23(23):14614. doi: 10.3390/ijms232314614 (PMC9741049; doi:10.3390/ijms232314614)
Supplement: Supplementary file 1 [file ijms-23-14614-s001.zip › ijms-2006847-supplementary.pdf]

cNTnC and fYTnC2, Genetically Encoded Green Calcium Indicators based on Troponin C from Fast Animals

Oksana M. Subach, Anna V. Vlaskina, Yuliya K. Agapova, Dmitriy A. Korzhenevskiy, Alena Y. Nikolaeva, Anna M. Varizhuk, Maksim F. Subach, Maxim V. Patrushev, Kiryl D. Piatkevich, Konstantin M. Boyko, and Fedor V. Subach

Supplementary Tables and Figures.

Table S1.

Table S2.

Table S3.

Figure S1.

Figure S2.

Figure S3.

Figure S4.

Figure S5.

Figure S6.

Figure S7.

Figure S8.

Figure S9.

Figure S10.

Figure S11.

Figure S12.

Supplementary Methods.

Table S1. Data collection and refinement statistics. *Highest resolution range is shown in parentheses.

PDB ID	5MWC
**Data collection**	
Diffraction source	ESRF (ID30A-3)
Detector	Eiger 4M
Wavelength (Å)	0.9677
Crystal-to-detector distance (mm)	170
Rotation range per image (°)	0.15
Total rotation range (°)	120
Space group	$P4_12_12$
Cell size	
a, b, c (Å)	82.10, 82.10, 157.47
α, β, γ (°)	90, 90, 90
Resolution (Å)	46.73-2.45 (2.50-2.45)*
R_{merge} (%)	6.2 (97.3)
<I>/<σ(I)>	23.9 (2.3)
Occupancy	99.7 (100)
Redundancy	8.9 (9.4)
$CC_{1/2}$	99.9 (73.3)
**Refinement**	
Reflections used in refinement	19409
R_{work} / $R_{free.}$	23.8 / 31.0
Number of non-hydrogen atoms	
Protein	4073
Ligands/ions	36
Solvent	46
Average B-factor	63.2
Protein	63.6
Ligands/ions	44.1
Solvent	46.3
R.m.s deviations	
Bond length (Å)	0.01
Angles (°)	1.86
Ramachandran plot	
Most favored (%)	95.9
Allowed (%)	2.9
Outliers (%)	1.2
MolProbity score	2.38

Table S2. *In vitro* properties of fYTnC2 compared to YTnC. [a] Data from [1]. Data marked with asterisk (*) were determined in this paper. [b] Quantum yields (QYs) were determined at pH 7.20. EGFP (QY=0.60 [2]) was used as the reference standard. [c] The extinction coefficients (ε) were determined by alkaline denaturation. [d] Brightness was calculated as a product of the quantum yield and extinction coefficient and normalized to the brightness of EGFP, which has an extinction coefficient of 56,000 $M^{-1}\cdot cm^{-1}$ and a quantum yield of 0.6 [2]. [e] The Hill coefficient is shown in brackets. [f] EGFP had a maturation half-time of 14 min. [g] Half-time to bleaching up to 50%. One-photon photobleaching was performed under a mercury lamp with drops in oil. Standard deviations are shown. EGFP had a photobleaching half-time of 305 ± 38 s.

<table>
<tr><th colspan="3" rowspan="3">Properties</th><th colspan="4">Proteins</th></tr>
<tr><th colspan="2">fYTnC2</th><th colspan="2">YTnC[a]</th></tr>
<tr><th>apo</th><th>sat</th><th>apo</th><th>sat</th></tr>
<tr><td colspan="3">Absorption maximum (nm)</td><td>414</td><td>494 (406)</td><td>413</td><td>495 (405)</td></tr>
<tr><td colspan="3">Excitation maximum (nm)</td><td>416 (500)</td><td>498 (418)</td><td>412, 501</td><td>502 (413)</td></tr>
<tr><td colspan="3">Emission maximum (nm)</td><td>516</td><td>518</td><td>514</td><td>516 (516)</td></tr>
<tr><td colspan="3">Quantum yield [b]</td><td>0.08±0.01
(0.09±0.02)</td><td>0.69±0.01
(0.24±0.02)</td><td>0.012</td><td>0.19 (0.03)</td></tr>
<tr><td colspan="3">ε (mM^{-1} cm^{-1}) [c]</td><td>98.1±9.4</td><td>46.6±2.8
(39.5±2.4)</td><td>28±2</td><td>29±3 (20±2)</td></tr>
<tr><td colspan="3">Brightness vs EGFP (%) [d]</td><td>23</td><td>96 (28)</td><td>1</td><td>17 (2)</td></tr>
<tr><td rowspan="3">ΔF/F</td><td rowspan="2">Purified protein</td><td>0 mM Mg^{2+}</td><td colspan="2">18±1</td><td colspan="2">10.6±0.4</td></tr>
<tr><td>1 mM Mg^{2+}</td><td colspan="2">17±1</td><td colspan="2">2.9±0.2</td></tr>
<tr><td colspan="2">HeLa cells</td><td colspan="2">4.0±1.5</td><td colspan="2">2.0±0.4</td></tr>
<tr><td colspan="3">pKa</td><td>9.1±0.1</td><td>6.4±0.1</td><td>5.2±0.1, 8.2±0.1</td><td>6.3±0.1</td></tr>
<tr><td rowspan="2">K_d (nM) [e]</td><td colspan="2">0 mM Mg^{2+}</td><td colspan="2">477±14
[n=2.6±0.2]</td><td colspan="2">223±10
[n=1.4±0.1]</td></tr>
<tr><td colspan="2">1 mM Mg^{2+}</td><td colspan="2">709±38
[n=2.0±0.2]</td><td colspan="2">410±19
[n=1.7±0.2]</td></tr>
<tr><td colspan="3">Maturation half-time (min) [f]</td><td colspan="2">4.6</td><td colspan="2">16</td></tr>
<tr><td colspan="3">Photobleaching half-time (s) [g]</td><td colspan="2">194±57</td><td colspan="2">193±36*</td></tr>
</table>

Table S3. List of primers.

Primer	Primer sequence (5′-3′)	Comment
**Neon-BglII-2**	gacAGATCTATGGTGAGCAAGGGCGAG	Use for mutagenesis of NTnC-derived mutants
**Neon-EcoRI-r2-r**	tcgaattcttacttgtacagctcgtccatg	
**Fw-LSSmOrange-BglII**	GTACGGCTCCAGGGCCTTCATCAAGCACCCCGCCGATATC	Use for mutagenesis of YTnC2-derived mutants
**Rv-GFP-EcoRI**	GATATCGGCGGGGTGCTTGATGAAGGCCCTGGAGCCGTAC	
**Fw-M146NNS**	GATGGCCCTGCGCCTGAAGGACGGCGGCCGCTACCTGGC	Use for mutagenesis of NTnC
**Rv-M146NNS**	GCCAGGTAGCGGCCGCCGTCCTTCAGGCGCAGGGCCATC	
**Rv-C224NNS**	GGCGGCCGCTACCTGGCAGACGTCAGGACCACCTACAAG	
**Fw-L240NNS**	CTTGTAGGTGGTCCTGACGTCTGCCAGGTAGCGGCCGCC	
**YTnC-NES3-r**	CAAGGCCAAGAAGCCCGTGCAGATGCCCGGCGCCTACAAC	Use for cloning of NTnC- and YTnC2-derived mutants into pAAV-CAG-NES plasmid

```
               1        10        20        30        40        50        60
               |        |         |         |         |         |         |
NTnC           MVSKGEEDNMASLPATHELHIFGSINGVDFDMVGQGSGNPNVGYEELNLKSTKGDLQFSP
NTnC2          MVSKGEEDNMASLPATHELHIFGSINGVDFDMVGQGSGNPNVGYEELNLKSTKGDLQFSP
iNTnC2         MVSKGEEDNMASLPATHELHIFGSINGADFDMVGQGSGNPNVGYEELNLKSTKGDLQFSP

                        70        80        90       100       110       120
                       ***         |         |         |         |         |
NTnC           WILVPHIGYGFHQYLPYPDGMS-PFQAAMVDGSGYQVHRTVQFEDGASLTVNYRYTYEGSH
NTnC2          WVLVPHIGYGFHQYLPYPDGMS-PFQAAMVDGSGYQVHRIVQFEDGASLTVNYRYTYEGSH
iNTnC2         WILVPHIGYGFHQYLPYPDGMS-PFQAAMVDGSGYQVHRTVQFEDGASLTVNYRYTYEGSH

                       130       140       150       160       170       180
                         |         |         |         |         |         |
NTnC           IKGEAQVKGTGFPADGPVMANSLT-AMVPSEEELSECFRTFDKDGDGFIDREEFGGIIRLT
NTnC2          IKGEAQVKGTGFPADGPVMANSLT-ARVPSEEGLSECFRTFDKDGDGFIDREEFGGIIRLA
iNTnC2         IKGEAQVKGTGFPADGPVMANSLT-AFAPSEEVLSECFRTFDKDGDGFLDREEFGGIIRLT

                       190       200       210       220       230       240
                         |         |         |         |         |         |
NTnC           GEQLTDEDPDEIFGDSDTDKNGRIDFDEFLKMVENVQLSMADWCRSKMACP-NDKTLISTL
NTnC2          GEQLTDEDPDEIFEDSDTDKDGRIDFDEFLKMVENVQLSMADWLRSKMACP-NDKTLISTD
iNTnC2         GEQLTDEDPDEIFEDSDTDKNGRIDFDEFLKMVENVQLSVADWYRSKMACP-NDKTLISTT

                       250       260       270       280       290       300
                         |         |         |         |         |         |
NTnC           KWSYTTGNGKRYRSTARTTYTFAKPMAANYLKNQP-MYVFRKTELKHSKTEMDFKEWQKAF
NTnC2          KWSYITGSGKRYRSTARTTYTFAKPMAANYLKNQP-MYVFRKIELKYSKTEMDFKEWQKAF
iNTnC2         KWSYTTGNGKRYRSTARTTYTFAKPMAANYLKNQP-MYVFRKTELEHSKTEMDFKEWQKAF

                       310
                         |
NTnC           TDVMGMDELYK
NTnC2          TDVMGMDELYK
iNTnC2         TDVMGMDELYK
```

Figure S1. Alignment of amino acid sequences for NTnC2, iNTnC2 and NTnC indicators. Amino acids internal to the β-can are marked by green color, amino acids of linkers are marked by yellow color, mutations in NTnC2 and iNTnC2 are highlighted by red color. Chromophore is marked by asterisks.

```
Acinonyx jubatus       1:SEEEIAECFRIFDRNADGYIIAEEIAEIFKASGEHVTDDEIESIMKDGDKNNDGRIDFDE  60
Calypte anna           1:SEEEIANCFRIFDRNADGFIIAEEIAEILRATGEQVTEEDIEDMMKDSDKNNDGRIDFDE  60
Crotalus adamanteus    1:SEEEIAECFRIFDRNADGFLIAEELVEIFRMSGEAVSEEEIQEIMRDGDKNNDGRIDFDE  60
Falco peregrinus       1:SEEEIANCFRIFDRNADGFIDIEELGEILRATGEHVTEEDIEDIMKDSDKNNDGRIDFDE  60
Harpegnathos saltator  1:MQEELKEAFRMYDREGNGYITTATLKEILAALDDKLTSSDLDGIIAEIDTDGSGTVDFDE  60
Myotis lucifugus       1:SEEEIAECFRIFDRNADGYIIAEELTEIFRASGEHVTEEEIESIMKDGDKNNDGRIDFDE  60
Toadfish               1:SEEELSECFRIFDKDGNGFIDREEFGDIIRLTGEQLTDEDPDEIFGDSDTDKNGRIDFDE  60

Acinonyx jubatus      61:FLKMMEGVQ                                                    69
Calypte anna          61:FLKMMEGVQ                                                    69
Crotalus adamanteus   61:FLKMMEGVQ                                                    69
Falco peregrinus      61:FLKMMEGVQ                                                    69
Harpegnathos saltator 61:FMEMMTGE-                                                    68
Myotis lucifugus      61:FLKMMEGVQ                                                    69
Toadfish              61:FLKMVENVQ                                                    69
```

Figure S2. Alignment of truncated troponins C from fast animals. Identical amino acid residues are marked by red frames.

```
fNTnC_library    1:MVSKGEEDNMASLPATHELHIFGSINGVDFDMVGQGSGNPNVGYEELNLKSTKGDLQFSP 60
aNTnC_library    1:MVSKGEEDNMASLPATHELHIFGSINGVDFDMVGQGSGNPNVGYEELNLKSTKGDLQFSP 60
cNTnC_library    1:MVSKGEEDNMASLPATHELHIFGSINGVDFDMVGQGSGNPNVGYEELNLKSTKGDLQFSP 60
CrNTnC_library   1:MVSKGEEDNMASLPATHELHIFGSINGVDFDMVGQGSGNPNVGYEELNLKSTKGDLQFSP 60
hNTnC_library    1:MVSKGEEDNMASLPATHELHIFGSINGVDFDMVGQGSGNPNVGYEELNLKSTKGDLQFSP 60
mNTnC_library    1:MVSKGEEDNMASLPATHELHIFGSINGVDFDMVGQGSGNPNVGYEELNLKSTKGDLQFSP 60
                   ************************************************************

fNTnC_library   61:WVLVPHIGYGFHQYLPYPDGMSPFQAAMVDGSGYQVHRIVQFEDGASLTVDYRYTYEGSH 120
aNTnC_library   61:WVLVPHIGYGFHQYLPYPDGMSPFQAAMVDGSGYQVHRIVQFEDGASLTVDYRYTYEGSH 120
cNTnC_library   61:WVLVPHIGYGFHQYLPYPDGMSPFQAAMVDGSGYQVHRIVQFEDGASLTVDYRYTYEGSH 120
CrNTnC_library  61:WVLVPHIGYGFHQYLPYPDGMSPFQAAMVDGSGYQVHRIVQFEDGASLTVDYRYTYEGSH 120
hNTnC_library   61:WVLVPHIGYGFHQYLPYPDGMSPFQAAMVDGSGYQVHRIVQFEDGASLTVDYRYTYEGSH 120
mNTnC_library   61:WVLVPHIGYGFHQYLPYPDGMSPFQAAMVDGSGYQVHRIVQFEDGASLTVDYRYTYEGSH 120
                   ************************************************************

fNTnC_library  121:IKGEAQVKGTGFPADGPVMANSLTARVXSEEELANCFRIFDRNADGFIDIEELGEILRAT 180
aNTnC_library  121:IKGEAQVKGTGFPADGPVMANSLTARVXSEEELAECFRIFDRNADGYIDAEELAEIFKAS 180
cNTnC_library  121:IKGEAQVKGTGFPADGPVMANSLTARVXSEEELANCFRIFDRNADGFIDAEELAEILRAT 180
CrNTnC_library 121:IKGEAQVKGTGFPADGPVMANSLTARVXSEEELAECFRIFDRNADGFLDAEELVEIFRMS 180
hNTnC_library  121:IKGEAQVKGTGFPADGPVMANSLTARVXMQEELKEAFRMYDREGNGYITTATLKEILAAL 180
mNTnC_library  121:IKGEAQVKGTGFPADGPVMANSLTARVXSEEELAECFRIFDRNADGYIDAEELTEIFRAS 180
                   ****************************..***...**..**...* .....* **  ..

fNTnC_library  181:GEHVTEEDIEDLMKDSDKNNDGRIDFDEFLKMMEGVQXSMADWLRSKMACPNDKTLISTD 240
aNTnC_library  181:GEHVTDDEIESLMKDGDKNNDGRIDFDEFLKMMEGVQXSMADWLRSKMACPNDKTLISTD 240
cNTnC_library  181:GEQVTEEDIEDMMKDSDKNNDGRIDFDEFLKMMEGVQXSMADWLRSKMACPNDKTLISTD 240
CrNTnC_library 181:GEAVSEEEIQELMRDGDKNNDGRIDFDEFLKMMEGVQXSMADWLRSKMACPNDKTLISTD 240
hNTnC_library  181:DDKLTSSDLDGIIAEIDTDGSGTVDFDEFMEMMTGE-XSMADWLRSKMACPNDKTLISTD 239
mNTnC_library  181:GEHVTEEEIESLMKDGDKNNDGRIDFDEFLKMMEGVQXSMADWLRSKMACPNDKTLISTD 240
                   .. .... .. .... *....*..*****..**.*....*********************

fNTnC_library  241:KWSYITGSGKRYRSTARTTYTFDKPMAANYLKNQPTYVFRKIELKYSKTEMDFKEWQKAF 300
aNTnC_library  241:KWSYITGSGKRYRSTARTTYTFDKPMAANYLKNQPTYVFRKIELKYSKTEMDFKEWQKAF 300
cNTnC_library  241:KWSYITGSGKRYRSTARTTYTFDKPMAANYLKNQPTYVFRKIELKYSKTEMDFKEWQKAF 300
CrNTnC_library 241:KWSYITGSGKRYRSTARTTYTFDKPMAANYLKNQPTYVFRKIELKYSKTEMDFKEWQKAF 300
hNTnC_library  240:KWSYITGSGKRYRSTARTTYTFDKPMAANYLKNQPTYVFRKIELKYSKTEMDFKEWQKAF 299
mNTnC_library  241:KWSYITGSGKRYRSTARTTYTFDKPMAANYLKNQPTYVFRKIELKYSKTEMDFKEWQKAF 300
                   ************************************************************

fNTnC_library  301:TDVMGMDELYK                                                  311
aNTnC_library  301:TDVMGMDELYK                                                  311
cNTnC_library  301:TDVMGMDELYK                                                  311
CrNTnC_library 301:TDVMGMDELYK                                                  311
hNTnC_library  300:TDVMGMDELYK                                                  310
mNTnC_library  301:TDVMGMDELYK                                                  311
                   ***********
```

Figure S3. Alignment of amino acid sequences of libraries of GECIs containing truncated troponins C of various fast animals and a derivative of the mNeonGreen fluorescent protein with a *trans*-chromophore. f — *Peregrine falcon* falcon, a — *Acinonyx jubatus* cheetah, c — *Calypte anna* hummingbird, m — *Myotis lucifugus* bat, Cr — *Crotalus adamanteus* rattlesnake, h — *Harpegnathos saltator* ant. Amino acids forming the chromophore are shown in green, linker amino acids in yellow, and amino acids in contact with calcium ions in blue.

```
aNTnCcis_lib   1:MVSKGEEENMASLPATHELHIFGSINGIDFDMVGQGTGNPNDGYEELNLKSTMGDLQFSP  60
cNTnCcis_lib   1:MVSKGEEENMASLPATHELHIFGSINGIDFDMVGQGTGNPNDGYEELNLKSTMGDLQFSP  60
fNTnCcis_lib   1:MVSKGEEENMASLPATHELHIFGSINGIDFDMVGQGTGNPNDGYEELNLKSTMGDLQFSP  60
                 ************************************************************

aNTnCcis_lib  61:WILVPHIGYGFHQYLPYPDGMSPFQAAMVDGSGYQVHRTMQFEDGASLTVNYRYTYEGSH 120
cNTnCcis_lib  61:WILVPHIGYGFHQYLPYPDGMSPFQAAMVDGSGYQVHRTMQFEDGASLTVNYRYTYEGSH 120
fNTnCcis_lib  61:WILVPHIGYGFHQYLPYPDGMSPFQAAMVDGSGYQVHRTMQFEDGASLTVNYRYTYEGSH 120
                 ************************************************************

aNTnCcis_lib 121:IKGEAQVEGTGFPADGLVMTNSLTAXXXSEEELAECFRIFDRNADGYIDAEELAEIFKAS 180
cNTnCcis_lib 121:IKGEAQVEGTGFPADGLVMTNSLTAXXXSEEELANCFRIFDRNADGFIDAEELAEILRAT 180
fNTnCcis_lib 121:IKGEAQVEGTGFPADGLVMTNSLTAXXXSEEELANCFRIFDRNADGFIDIEELGEILRAT 180
                 **********************************.***********.**.***.**..*.

aNTnCcis_lib 181:GEHVTDDEIESLMKDGDKNNDGRIDFDEFLKMMEGVQXXXADWCVSKKTYPNDKTIVSTL 240
cNTnCcis_lib 181:GEQVTEEDIEDMMKDSDKNNDGRIDFDEFLKMMEGVQXXXADWCVSKKTYPNDKTIVSTL 240
fNTnCcis_lib 181:EEHVTEEDIEDLMKDSDKNNDGRIDFDEFLKMMEGVQXXXADWCVSKKTYPNDKTIVSTL 240
                 .*.**...**..***.********************************************

aNTnCcis_lib 241:KWAFITDNGKRYRSTARTTYTFAKPMAANYLKNQPMYVFRKTELKHSKTELNFKEWQKAF 300
cNTnCcis_lib 241:KWAFITDNGKRYRSTARTTYTFAKPMAANYLKNQPMYVFRKTELKHSKTELNFKEWQKAF 300
fNTnCcis_lib 241:KWAFITDNGKRYRSTARTTYTFAKPMAANYLKNQPMYVFRKTELKHSKTELNFKEWQKAF 300
                 ************************************************************

aNTnCcis_lib 301:TDVMGMDELYK                                                  311
cNTnCcis_lib 301:TDVMGMDELYK                                                  311
fNTnCcis_lib 301:TDVMGMDELYK                                                  311
                 ***********
```

Figure S4. Alignment of amino acid sequences of GECIs libraries containing truncated troponins C from various fast animals and a derivative of the mNeonGreen fluorescent protein with a *cis*-chromophore. — *Peregrine falcon* falcon, a — *Acinonyx jubatus* cheetah, c — *Calypte anna* hummingbird. Amino acids forming the chromophore are shown in green, linker amino acids in yellow, and amino acids in contact with calcium ions in blue.

```
aNTnCtrans      1:MVSKGEEDNMASLPATHELHIFGSINGVDFDMVGQGSGNPNVGYEELNLRSTKGDLQFSP  60
aNTnC_library   1:MVSKGEEDNMASLPATHELHIFGSINGVDFDMVGQGSGNPNVGYEELNLKSTKGDLQFSP  60
cNTnCtrans      1:MVSKGEEDNMASLPATHELHIFGSINGVDFDMVGLGSGNPNVGYEELNLKSTKGDLQFSP  60
cNTnC_library   1:MVSKGEEDNMASLPATHELHIFGSINGVDFDMVGQGSGNPNVGYEELNLKSTKGDLQFSP  60
fNTnCtrans      1:MVSKGEEDNMASLPATHELHIFGSINGVDFDMVGQGSGNPIVGYEELNLKSTKGDLQFSP  60
fNTnC_library   1:MVSKGEEDNMASLPATHELHIFGSINGVDFDMVGQGSGNPNVGYEELNLKSTKGDLQFSP  60
                  **********************************.*****.*********.*********

aNTnCtrans     61:WVLVPHIGYGFHQYLPYPDGMSPFQAAMVDGSGYQVHRIVQFEDGASLTVDYRYTYEGSH 120
aNTnC_library  61:WVLVPHIGYGFHQYLPYPDGMSPFQAAMVDGSGYQVHRIVQFEDGASLTVDYRYTYEGSH 120
cNTnCtrans     61:WVLVPHIGYGFHQYLPYPDGMSPFQAAMVDGSGYQVHRIVQFEDGASLTVDYRYTYEGSH 120
cNTnC_library  61:WVLVPHIGYGFHQYLPYPDGMSPFQAAMVDGSGYQVHRIVQFEDGASLTVDYRYTYEGSH 120
fNTnCtrans     61:WVLVPHIGYGFHQYLPYPDGMSPFQAAMVDGSGYQVHRIVLFEDGASLTVDYRYTYEGSH 120
fNTnC_library  61:WVLVPHIGYGFHQYLPYPDGMSPFQAAMVDGSGYQVHRIVQFEDGASLTVDYRYTYEGSH 120
                  ****************************************.*******************

aNTnCtrans    121:IKGEAQVKGTGFPADGPVMANSLTARVQSEEELAECFCIFDGNADGYIDAEEMAEIFKAS 180
aNTnC_library 121:IKGEAQVKGTGFPADGPVMANSLTARVXSEEELAECFRIFDRNADGYIDAEELAEIFKAS 180
cNTnCtrans    121:IKGEAQVKGTGFPADGPVMANSLTARVMSEVELANCFRIFDRNADGFIDAEELAEILRTT 180
cNTnC_library 121:IKGEAQVKGTGFPADGPVMANSLTARVXSEEELANCFRIFDRNADGFIDAEELAEILRAT 180
fNTnCtrans    121:IKGEAQVKGTGFPADGPVMANSLTARVLSEEELANSFRICDRNADGFIDIEELGEILRAT 180
fNTnC_library 121:IKGEAQVKGTGFPADGPVMANSLTARVXSEEELANCFRIFDRNADGFIDIEELGEILRAT 180
                  *************************** **.***..*.*.*.****.**.**..**....

aNTnCtrans    181:GEHVTDDEIESLMKDGDKNNDGRIDFDEFLKMMEGVQMSMANWLRSKMACPNDKTLISTD 240
aNTnC_library 181:GEHVTDDEIESLMKDGDKNNDGRIDFDEFLKMMEGVQXSMADWLRSKMACPNDKTLISTD 240
cNTnCtrans    181:GEQVTEEDIEDMMKDSDKNNDGRIDFDEFLKMTEGVQLPMVDWLRSKMACPNDKTLISTD 240
cNTnC_library 181:GEQVTEEDIEDMMKDSDKNNDGRIDFDEFLKMMEGVQXSMADWLRSKMACPNDKTLISTD 240
fNTnCtrans    181:EERVTEEDIEDLMMDSDKNNDGRIDFDEFLKMMEGAQLSMADWLRSKMACPNDKTLISTD 240
fNTnC_library 181:GEHVTEEDIEDLMKDSDKNNDGRIDFDEFLKMMEGVQXSMADWLRSKMACPNDKTLISTD 240
                  .* **...**..*.*.****************.**.* .*..******************

aNTnCtrans    241:KWSYITGSGKRYRSTARTTYTFGKPMAANYLKNQPTYVLRKVELKYSKTEMDFKEWQKAF 300
aNTnC_library 241:KWSYITGSGKRYRSTARTTYTFDKPMAANYLKNQPTYVFRKIELKYSKTEMDFKEWQKAF 300
cNTnCtrans    241:KWSYITGSGNRYRATARTTYTFDKPMAANYLKNQPTYVSRKIELKYFKTEMDFIEWQKAF 300
cNTnC_library 241:KWSYITGSGKRYRSTARTTYTFDKPMAANYLKNQPTYVFRKIELKYSKTEMDFKEWQKAF 300
fNTnCtrans    241:KWSYITGSGKRYRTTARTTYTFDKPMAANYLKNQPTYVFRKIELKYSKTEMDFKEWQKAF 300
fNTnC_library 241:KWSYITGSGKRYRSTARTTYTFDKPMAANYLKNQPTYVFRKIELKYSKTEMDFKEWQKAF 300
                  *********.***.********.***************.**.****.******.******

aNTnCtrans    301:TDVMGMDELYK                                                 311
aNTnC_library 301:TDVMGMDELYK                                                 311
cNTnCtrans    301:TDVVGMDELYK                                                 311
cNTnC_library 301:TDVMGMDELYK                                                 311
fNTnCtrans    301:TDAMGMDELYK                                                 311
fNTnC_library 301:TDVMGMDELYK                                                 311
                  **..*******
```

Figure S5. Alignment of amino acid sequences for developed indicators with *trans*-chromophore and libraries of calcium indicators containing truncated troponins C of various fast animals and a derivative of mNeonGreen fluorescent protein with *trans*-chromophore. f — *Peregrine falcon* falcon, a — *Acinonyx jubatus* cheetah, c — *Calypte anna* hummingbird. Linker amino acids are in red, chromophore-forming amino acids are in green, amino acids in contact with calcium ions are in blue, and amino acid substitutions in the resulting mutants compared to the original libraries are in pink, amino acids of linkers in the resulting mutants after overlap mutagenesis are in yellow.

```
aNTnCcis            1:MVSKGEEENMASLPATHELHIFGSINGIDFDMVGQGTGNPNDGYEELNLKSTMGDLQFSP 60
aNTnCcis_lib        1:MVSKGEEENMASLPATHELHIFGSINGIDFDMVGQGTGNPNDGYEELNLKSTMGDLQFSP 60
cNTnCcis            1:MVSKGEEENMASLPATHELHIFGSINGIDFDMVGQGTGNPNDGYEELNLKSTMGDLQFSP 60
cNTnCcis_lib        1:MVSKGEEENMASLPATHELHIFGSINGIDFDMVGQGTGNPNDGYEELNLKSTMGDLQFSP 60
fNTnCcis            1:MVSKGEEENMASLPATHELHIFGSINGIDFDMVGQGTGNPNDGYEELSLKSTMGELQFSP 60
fNTnCcis_lib        1:MVSKGEEENMASLPATHELHIFGSINGIDFDMVGQGTGNPNDGYEELNLKSTMGDLQFSP 60
                      ***********************************************.******.*****

aNTnCcis           61:WILVPHIGYGFHQYLPYPDGMSPFQAAMVDGSGYQVHRTMQFEDGATLTVNYRYTYEGSH 120
aNTnCcis_lib       61:WILVPHIGYGFHQYLPYPDGMSPFQAAMVDGSGYQVHRTMQFEDGASLTVNYRYTYEGSH 120
cNTnCcis           61:WILVPHIGYGFHQYLPYPDGMSPFQAAMVDGSGYQVHRTMQFEDGASLTVNYRYAYEGSH 120
cNTnCcis_lib       61:WILVPHIGYGFHQYLPYPDGMSPFQAAMVDGSGYQVHRTMQFEDGASLTVNYRYTYEGSH 120
fNTnCcis           61:CILVPHIGYGFHQYLPYPDGMSPFQAAMVDGSGYQVHRTMQFEDGASLTVDYRYTYEGSH 120
fNTnCcis_lib       61:WILVPHIGYGFHQYLPYPDGMSPFQAAMVDGSGYQVHRTMQFEDGASLTVNYRYTYEGSH 120
                      .*********************************************.***.***.*****

aNTnCcis          121:IKGEAQVEGTGFPADGPVMTNSLTAKIFSEEELAECFSIFDGNADGYIDAEELAEIFKAS 180
aNTnCcis_lib      121:IKGEAQVEGTGFPADGLVMTNSLTAXXXSEEELAECFRIFDRNADGYIDAEELAEIFKAS 180
cNTnCcis          121:IKGEAQVEGTGFPADGPVMTNSLTAIIYSEEELAICFRIFDRNADGFIDAEELAEILRAT 180
cNTnCcis_lib      121:IKGEAQVEGTGFPADGLVMTNSLTAXXXSEEELANCFRIFDRNADGFIDAEELAEILRAT 180
fNTnCcis          121:IVGEAQVEGTGFPADGPVMANSLTAISTSEEELANCFRICDRNADGFIDIEELGEILRAT 180
fNTnCcis_lib      121:IKGEAQVEGTGFPADGLVMTNSLTAXXXSEEELANCFRIFDRNADGFIDIEELGEILRAT 180
                      *.************** **.*****   ******.**.*.*.****.**.***.**..*.

aNTnCcis          181:GEHVTDDEIESLMKDGDKNNDGRIDFDEFLKMMEGEQVVPTDWCVSKKTYPNDKTIVSTL 240
aNTnCcis_lib      181:GEHVTDDEIESLMKDGDKNNDGRIDFDEFLKMMEGVQXXXADWCVSKKTYPNDKTIVSTL 240
cNTnCcis          181:GVQVTEEDIEDMMKDSDKNNDGRIDFDEFLKMMEGVQQVPADWCVSKKTYPNDKTIVSTL 240
cNTnCcis_lib      181:GEQVTEEDIEDMMKDSDKNNDGRIDFDEFLKMMEGVQXXXADWCVSKKTYPNDKTIVSTL 240
fNTnCcis          181:EERVTEEDIEDLMKDSDKNNDGRIDFDEFLKMMEGAQGVIADWCVSQKTYPNDKTIVSTL 240
fNTnCcis_lib      181:EEHVTEEDIEDLMKDSDKNNDGRIDFDEFLKMMEGVQXXXADWCVSKKTYPNDKTIVSTL 240
                      ...**...**..***.*******************.*   .*****.*************

aNTnCcis          241:KWALITDNGKRYRSTARTTYTFAKPMAANYLKNQPMYVFRKTELKHFKTELNFKEWQKAF 300
aNTnCcis_lib      241:KWAFITDNGKRYRSTARTTYTFAKPMAANYLKNQPMYVFRKTELKHSKTELNFKEWQKAF 300
cNTnCcis          241:KWAFITDNGKRYRSTARTTYTFAKPMAANYLKNQPMYVFRKTELKHSKTELSFKEWQKAF 300
cNTnCcis_lib      241:KWAFITDNGKRYRSTARTTYTFAKPMAANYLKNQPMYVFRKTELKHSKTELNFKEWQKAF 300
fNTnCcis          241:KWVFITDDGKRYRSTARTTYSFAKPMAANYLKNQPMYVFRKTELKHFKTELNFKEWQKAF 300
fNTnCcis_lib      241:KWAFITDNGKRYRSTARTTYTFAKPMAANYLKNQPMYVFRKTELKHSKTELNFKEWQKAF 300
                      **..***.************.*************************.****.********

aNTnCcis          301:TDVMGMDELYK                                                 311
aNTnCcis_lib      301:TDVMGMDELYK                                                 311
cNTnCcis          301:TDVMGMDELYK                                                 311
cNTnCcis_lib      301:TDVMGMDELYK                                                 311
fNTnCcis          301:TDVMGMDELYK                                                 311
fNTnCcis_lib      301:TDVMGMDELYK                                                 311
                      ***********
```

Figure S6. Alignment of the amino acid sequences of the developed indicators with a cis chromophore and libraries of calcium indicators containing truncated troponins C from various fast animals and a derivative of the mNeonGreen fluorescent protein with a cis chromophore. f — *Peregrine falcon* falcon, a — *Acinonyx jubatus* cheetah, c— *Calypte anna* hummingbird. Chromophore-forming amino acids are in green, linker amino acids are in yellow, amino acids in contact with calcium ions are in blue, and amino acid substitutions in the resulting mutants compared to the original libraries are in pink.

```
aYTnC   1: MVSKGEELFTGVVPILVEMVGDVNGHRFSVSGEGEGIATYGMLTLKLICTTGELPVPWPT  60
cYTnC   1: MVSKGEELFTGVVPILVEMVGDVNGHRFSVSGEGEGIATYGMLTLKLICTTGELPVPWPT  60
fYTnC   1: MVSKGEELFTGVVPILVEMVGDVNGHRFSVSGEGEGIATYGMLTLKLICTTGELPVPWPT  60
           ************************************************************

aYTnC  61: LVTTLGYGVACFARYPDHMKQHDFFKSAMPEGYVQERTIFFKGDGYYKTRAEVKFEGDTL 120
cYTnC  61: LVTTLGYGVACFARYPDHMKQHDFFKSAMPEGYVQERTIFFKGDGHYKTRAEVKFEGDTL 120
fYTnC  61: LVTTLGYGVACFARYPDHMKQHDFFKSAMPEGYVQERTIFFKGDGHYKTRAEVKFEGDTL 120
           *********************************************.**************

aYTnC 121: VNRIELKGFDFREDGNILGHKLGYNFDLSEEELAECFCIFDGNADGYIDAEEMAEIFKAS 180
cYTnC 121: VNRIELKGFDFREDGNILGHKLGYNFDLSEEELAICFRIFDRNADGFIDAEELAEILRAT 180
fYTnC 121: VNRIELKGFDFREDGNILGHKLGYNFDLSEEELANSFRICDRNADGFIDIEELGEILRAT 180
           **********************************..*.*.*.****.**.**..**..*.

aYTnC 181: GEHVTDDEIESLMKDGDKNNDGRIDFDEFLKMMEGVQGINSHNVYITADKQKNGVKAHFE 240
cYTnC 181: GVQVTEEDIEDMMRDSDENNDGRIDFDEFLKMKEGMQGINSHNVYITADKQKNGVKAHFE 240
fYTnC 181: EERVTEEDIEDLMMDSDKNNDGRIDFDEFLKMMEGALGINSHNVYITADKQKNGVKAHFE 240
           ...**...**..*.*.*.**************.**..***********************

aYTnC 241: IRHNLEDGSVQLADHYQLNTPIGDGPVLLPDNHYLRHQSALSKDPNEKRDHMVLQEFVTA 300
cYTnC 241: IRHNLEDGSVQLADHYQLNTPIGDGPVLLPDNHYLRHQSALSKDPNEKRDHMVLQEFVTA 300
fYTnC 241: IRHNLEDGSVQLADHYQLNTPIGDGPVLLPDNHYLRHQSALSKDPNEKRDHMVLQEYVTA 300
           ********************************************************.***

aYTnC 301: AGITHGMDELYK 312
cYTnC 301: AGITHGMDELYK 312
fYTnC 301: AGITHGMDELYK 312
           ************
```

Figure S7. Alignment of the amino acid sequences of the developed indicators containing truncated troponins C from various fast animals and a derivative of the YFP fluorescent protein. f — *Peregrine falcon* falcon, a — *Acinonyx jubatus* cheetah, c— *Calypte anna* hummingbird. Chromophore-forming amino acids are shown in green, linker amino acids in yellow, amino acids in contact with calcium ions in blue, and amino acid substitutions in the resulting mutants compared to the original libraries in pink.

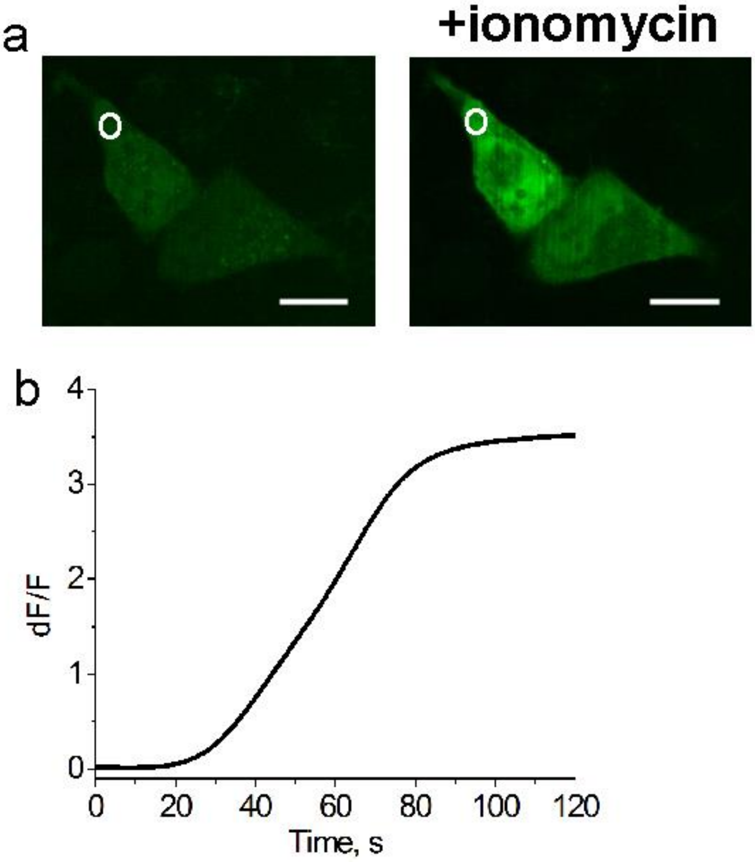

Figure S8. Response of fYTnC2 to ionomycin-induced increase of Ca^{2+} concentration in HeLa Kyoto cells. Top, confocal images of HeLa cells expressing fYTnC2 in green fluorescence channel (with 488 nm excitation) before and after ionomycin addition. Bottom, the graph shows the changes in green fluorescence as a result of the addition of 5 μM ionomycin. The graph illustrates changes in green fluorescence in the areas indicated with white circles. Scale bars, 100 μm.

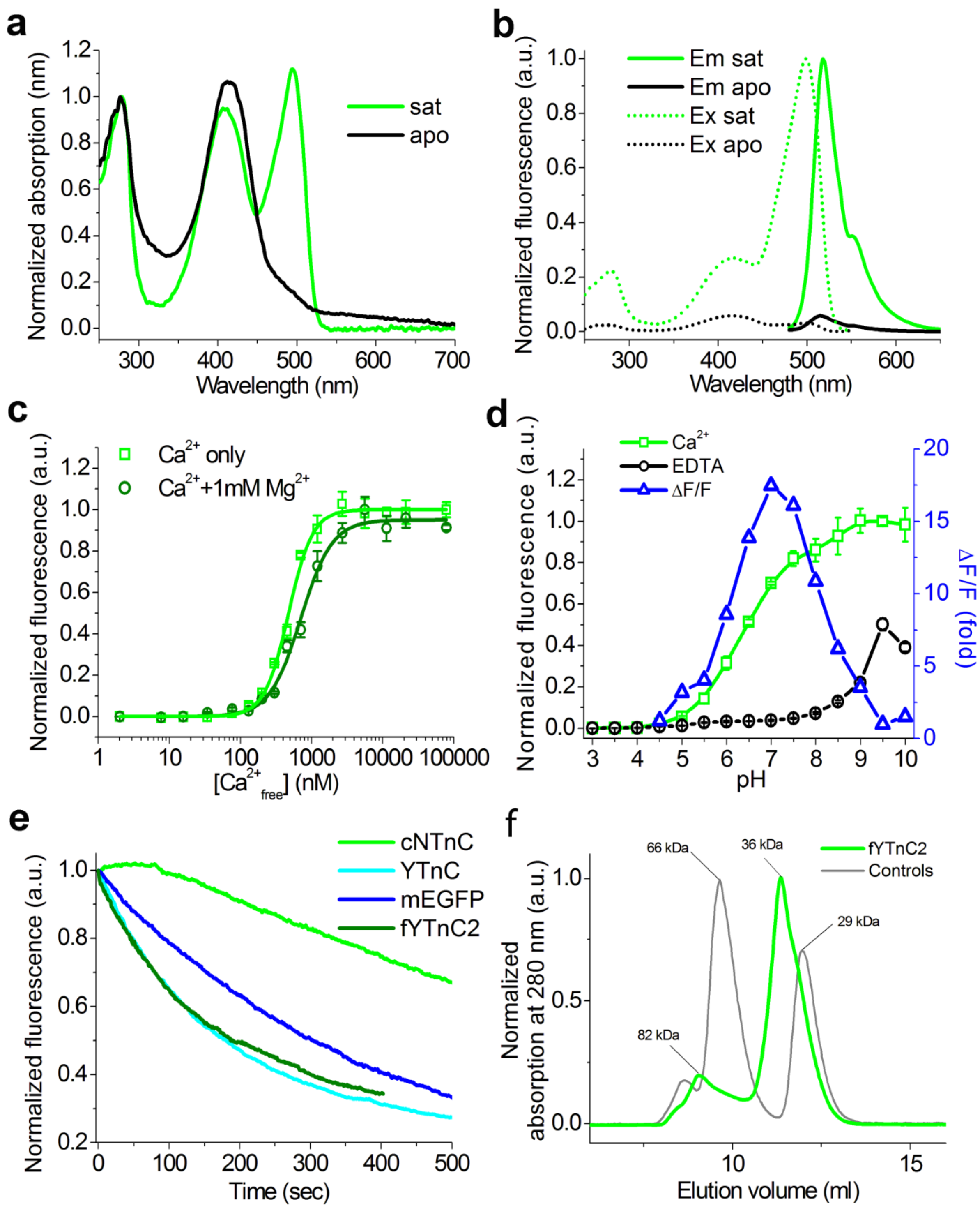

Figure S9. Properties fYTnC2 in vitro. (a) Absorbance spectra of fYTnC2 in Ca^{2+}-free (apo) and Ca^{2+}-bound (sat) states. (b) Excitation and emission spectra of fYTnC2 in Ca^{2+}-free and Ca^{2+}-bound states. (c) Ca^{2+} titration curves for fYTnC2 in the absence or presence of 1 mM $MgCl_2$. (d) Intensity and dynamic range of fYTnC2 as a function of pH. The dF/F fluorescence response (fold) at each pH value was determined as the ratio of fYTnC2 fluorescence intensity in the absence of Ca^{2+} to that in the presence of Ca^{2+}. (e) Photobleaching curves for fYTnC2, cNTnC and YTnC in the Ca^{2+}-bound states and for mEGFP. (f) Fast protein liquid chromatography of fYTnC2. fYTnC2 was eluted in 20 mM Tris-HCl (pH 7.80) and 200 mM NaCl buffer. The molecular weight of fYTnC2 was calculated from a linear regression of the dependence of the logarithm of the control molecular weights vs. elution volume (**Figure S7**). Error represents the standard error of the estimate for the average of three records.

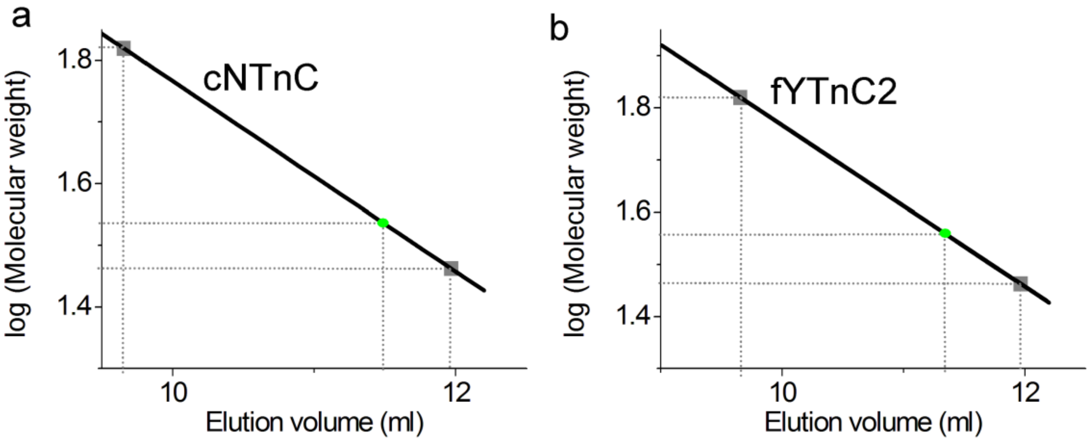

Figure S10. Fast protein liquid chromatography of cNTnC and fYTnC2 protein. cNTnC (a) and fYTnC2 (b) were eluted in 20 mM Tris-HCl (pH 7.80) and 200 mM NaCl buffer supplemented with 5 mM $CaCl_2$ as shown in Figures 8f and S6f. The molecular weight of cNTnC and fYTnC2 were calculated from the dependence of logarithm of control molecular weights vs. elution volume.

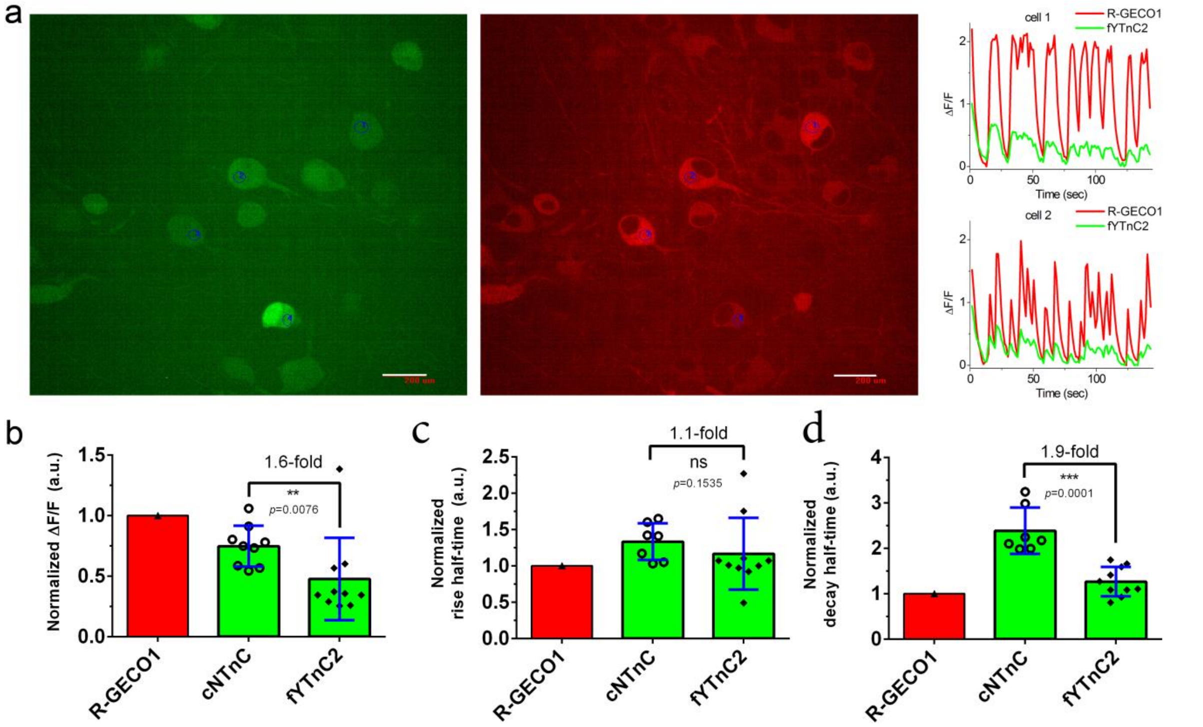

Figure S11. Spontaneous neuronal activity of fYTnC2 co-expressed with R-GECO1 in neuronal cultures. (a) Left, confocal images of green and red fluorescence with excitation at 488 and 561 nm of HeLa cells co-expressing fYTnC2 with R-GECO1. Scale bars, 50 μm. Right, the graphs show the changes in green and red fluorescence in two cells in the areas indicated with blue circles. (b) The dF/F responses for cNTnC and fYTnC2 were calculated according to the dF/F response of R-GECO1 in the same cell. (c,d) The rise (c) and decay (d) half-times for cNTnC and fYTnC2. (b–d) Error bars are the standard deviations across 7–10 cells. Ns, not significant, $p>0.05$. **, p-value is from 0.001 to 0.01. ***, p-value is from 0.0001 to 0.001.

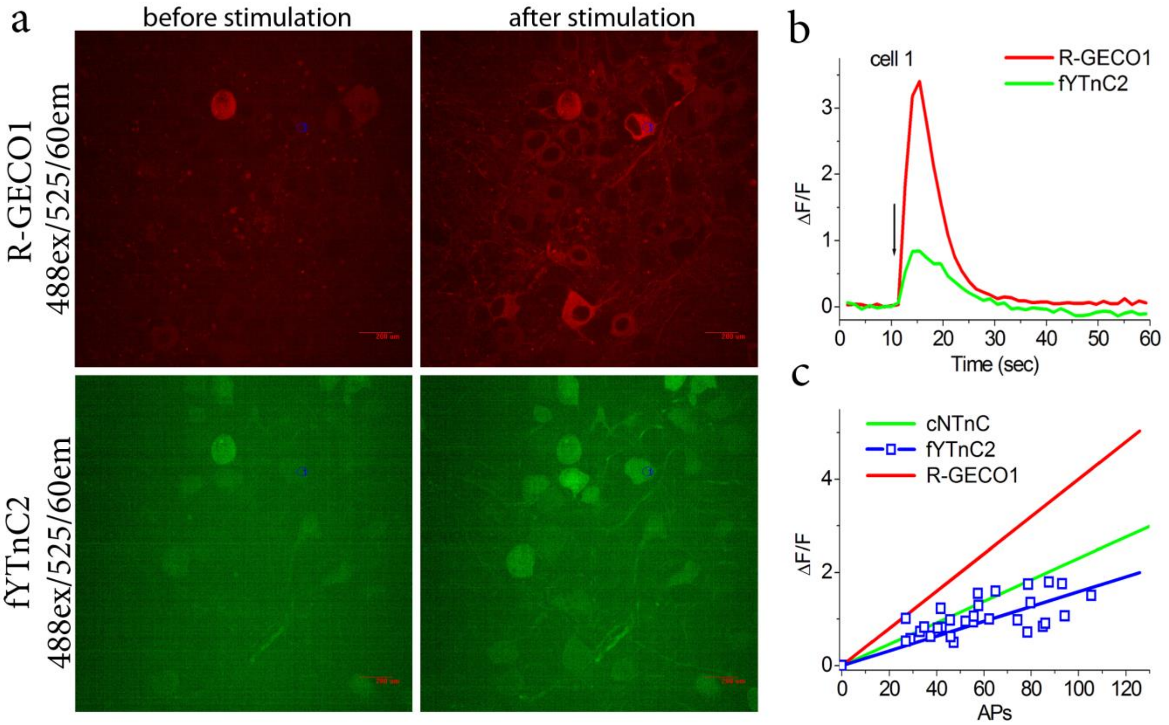

Figure S12. Comparison of the responses of green fYTnC2 and red R-GECO1 indicators to the external field stimulation of neurons co-expressing the GECIs in dissociated neuronal cultures. Neuronal cultures co-expressing the NES-fYTnC2 and NES-R-GECO1 indicators were imaged and stimulated on DIV 21–22th. Neuronal cultures were transduced on DIV fourth with a mixture of rAAVs carrying NES-fYTnC2 and NES-R-GECO1. (a) Confocal images of neuronal culture co-expressing the NES-cNTnC and NES-R-GECO1 indicators before (left) and after (right) electrical stimulation. Scale bar, 50 μm. (b) The graph illustrates ΔF/F changes in the green and red fluorescence of the fYTnC2 and R-GECO1 indicators in response to an electrical field stimulation. The changes on the graph correspond to the area indicated on panel a as a white circle. (c) The dependence of ΔF/F responses for the fYTnC2 indicator vs. the number of action potentials (APs). A number of APs was determined according to the ΔF/F response of the R-GECO1 indicator (0.04 per 1 AP) co-expressed in the same cell and assuming that response's linearity in the examined AP range (linear fitting for fYTnC2 had R2 value of 0.68211). The dependences of ΔF/F responses on APs for cNTnC and R-GECO1 were added for comparison.

Supplementary Methods.

Preparative protein purification.

For preparative protein purification for X-ray crystallography, the bacterial cells expressing the NTnC protein with a His-tag and Tobacco Etch Virus (TEV) protease cleavage site were harvested by centrifugation for 20 min at 5,000×*rpm* and 4 °C using Avanti J-E centrifuge (Beckman Coulter, USA). The pellet was further resuspended in 40 mM Tris(tris(hydroxymethyl)aminomethane)-HCl buffer, pH 7.5 supplemented with 400 mM NaCl, 10 mM Imidazole, 0.2% Triton X-100, and 1 mM PMSF (phenylmethylsulfonyl fluoride) (7 ml per 1 g of the cells), and disrupted by sonication (2 sec pulse-6-sec pause, 45% amplitude, for total time of 5 minutes). The crude cell extract was centrifuged for 30 min at 28,000× *g* and 4 °C using Avanti J-E centrifuge (Beckman Coulter, USA). The supernatant was applied to a 5 ml Ni-NTA Superflow column (Qiagen, Hilden, Germany) equilibrated with the binding buffer (40 mM Tris-HCl, pH 7.5, containing 400 mM NaCl, 10 mM imidazole, and 0.1% (*v/v*) Triton X-100). The column was further washed using the same binding buffer without Triton X-100 followed by second washing using binding buffer in the absence of Triton X-100, supplemented with 40 mM imidazole. Protein elution was performed using the same binding buffer without Triton X-100 and supplemented with 300 mM imidazole. 1mM DTT (dithiothreitol) (final concentration) and 1mM EDTA (ethylenediaminetetraacetic acid) (final concentration) were added to the protein solution and mixed with TEV protease (1 mg per 10 mg of the protein). The final mix was dialyzed for 16 hours in dialysis buffer (40 mM Tris-HCl, pH 7.8, 400 mM NaCl, 5 mM Imidazole, 2 mM BME (2-mercaptoethanol), 1 mM EDTA), at +4 °C (monitoring of the His-tag cleavage was conducted using 12 % SDS-PAGE (sodium dodecyl sulphate–polyacrylamide gel electrophoresis)). After dialysis, the protein solution was applied to a Ni-NTA Superflow column (Qiagen, EU), equilibrated with dialysis buffer; TEV protease and cleaved His-tag were absorbed by the Ni-NTA Superflow column (Qiagen, EU) and the flow-through was concentrated till 2.3 ml volume using a 10 kDa cutoff centrifugal filter device (Millipore, Burlington, MA, USA). The buffer of the concentrated protein was exchanged for the 20 mM Tris pH 7.5, 5mM $CaCl_2$, 5% (v/v) glycerol buffer using PD-10 column (GE Healthcare, Sweden). The concentrated protein was further applied to a 1 ml ResourceQ column (GE Healthcare, Sweden) equilibrated with the same 20 mM Tris pH 7.5, 5mM $CaCl_2$, 5% (v/v) glycerol buffer. Recombinant NTnC was eluted using a linear gradient from 0 to 1M NaCl; protein eluted by one peak at 250 mM NaCl. Protein concentration was measured for each of the fractions individually using Bicinchoninic Acid Protein Assay Kit (Sigma-Aldrich, Saint Louis, USA), BSA (bovine serum albumin) protein standard (P0914-5AMP, Sigma-Aldrich, Saint Louis, USA) solution as standard. Total protein yield was 4.8 mg. The fractions were loaded onto the Superdex 75 10/300 GL column (GE Healthcare, Sweden), in 20 mM Tris-HCl pH 8.0, 5mM $CaCl_2$, , 250 mM NaCl buffer. In both cases, 15.4-17.5 ml fractions (corresponding to monomer) were collected and concentrated using a 10 kDa cutoff centrifugal filter device (Millipore, Burlington, MA, USA) till 15 mg/ml concentration for the crystallization. Protein purity at each step was monitored using 12% SDS-PAGE. Chromatography was performed using ÄKTA prime plus and ÄKTA explorer 100 systems (GE Healthcare, Sweden).

Mammalian cell imaging

Transient transfection of the HeLa Kyoto cells was performed in 24-well format using lipofectamine reagent according to the manufacture's protocol. Cells were cultured using DMEM medium supplemented with 10% FBS, Glutamine, 50 U/ml penicillin, and 50 U/ml streptomycin, at 37 degrees and 5% CO_2. HeLa cell cultures were imaged 24 h after the transient transfection using a laser spinning-disk

Andor XDi Technology Revolution multi-point confocal system (Andor Technology, UK) equipped with an inverted Nikon Eclipse Ti-E/B microscope (Nikon Instruments, Japan), a 75 W mercury-xenon lamp (Hamamatsu, Japan), a 60× oil immersion objective NA 1.4 (Nikon, Japan), a 16-bit Neo sCMOS camera (Andor Technology, UK), laser module Revolution 600 (Andor Technology, UK), spinning-disk module Yokogawa CSU-W1 (Andor Technology, UK). The green and red fluorescence were acquired using the 488 nm or 561 nm lasers, confocal dichroic mirror 405/488/561/640 and filter wheel, 525/50 or 617/73 emission filters, respectively. During imaging cells were incubated at 37 degrees and 5% CO_2 using a cage incubator (Okolab, Italy).

Imaging of neuronal cultures

Dissociated neuronal cultures were isolated from the C57BL/6 mice at postnatal days 0–1 and were grown on a 24-well cell imaging black plate with a glass bottom; then, the tissue cultures were treated (Eppendorf, Hamburg, Germany) in Neurobasal Medium A (GIBCO, Paisley, Scotland, UK) supplemented with 2% B27 Supplement (GIBCO, Paisley, Scotland, UK), 0.5 mM glutamine (GIBCO, Paisley, Scotland, UK), 50 U/mL penicillin, and 50 g/mL streptomycin (GIBCO, Paisley, Scotland, UK). On the fourth day in vitro, neuronal cultures were transduced with a mixture of rAAV viral particles (DJ serotype) carrying AAV-CAG-NES-R-GECO1 and AAV-CAG-NES-cNTnC, AAV-CAG-NES-aNTnC, AAV-CAG-NES-fNTnC, AAV-CAG-NES-cYTnC, AAV-CAG-NES-aYTnC, or AAV-CAG-NES-fYTnC. The cells were imaged using an Andor XDi Technology Revolution multi-point confocal system on DIV 15 (spontaneous activity at 37°C, 5% carbon dioxide) and 21–22 (electrical field stimulation at r.t.).

Stimulation of neuronal cultures was performed using a self-built electrical system described earlier [1]. In this step, 300 voltage pulses of a 1 ms duration (0.5 ms negative phase, 0.5 ms interphase, and 0.5 ms positive phase) at a 87 Hz frequency with an amplitude of +-70 V were applied to the neuronal cultures in 24-well plates through iridium electrodes with a 5 mm gap. Then, 10 μM cyanquixaline (6-cyano-7-nitroquinoxaline-2,3-dione) (CNQX) and 100 μM (2R)-amino-5-phosphonovaleric acid (APV or AP5) were added before stimulation to block spontaneous neuronal activity. To quantify the fluorescence intensity, the background noise determined from the adjacent cell-free area was subtracted from mean fluorescence intensity value for the cytosolic sub-region of the cell of the similar area. The rise and decay half-times were calculated as time difference between time point corresponding to the calcium spike maximum and time points at half-maximum on the left and right edges of the spike, respectively.

References

1. Barykina, N. V.; Doronin, D. A.; Subach, O. M.; Sotskov, V. P.; Plusnin, V. V.; Ivleva, O. A.; Gruzdeva, A. M.; Kunitsyna, T. A.; Ivashkina, O. I.; Lazutkin, A. A.; Malyshev, A. Y.; Smirnov, I. V.; Varizhuk, A. M.; Pozmogova, G. E.; Piatkevich, K. D.; Anokhin, K. V.; Enikolopov, G.; Subach, F. V., NTnC-like genetically encoded calcium indicator with a positive and enhanced response and fast kinetics. *Sci Rep* **2018,** 8, (1), 15233.
2. Tsien, R. Y., The green fluorescent protein. *Annu Rev Biochem* **1998,** 67, 509-44.
